# Proficiency of data interpretation: identification of signaling SNPs/specific loci for coronary artery disease

**DOI:** 10.1093/database/bax078

**Published:** 2017-10-31

**Authors:** Asma N Cheema, Samantha L Rosenthal, M Ilyas Kamboh

**Affiliations:** 1 Atta-Ur-Rahman School of Applied Biosciences, National University of Sciences & Technology, Islamabad, Pakistan; 2 Department of Pathology, University Medical & Dental College, The University of Faisalabad, Faisalabad, Pakistan and; 3 Department of Human Genetics, University of Pittsburgh, Pittsburgh, PA, USA

## Abstract

Coronary artery disease (CAD) is a complex disorder involving both genetic and non-genetic factors. Genome-wide association studies (GWAS) have identified hundreds of single nucleotides polymorphisms (SNPs) tagging over > 40 CAD risk loci. We hypothesized that some non-coding variants might directly regulate the gene expression rather than tagging a nearby locus. We used RegulomeDB to examine regulatory functions of 58 SNPs identified in two GWAS and those SNPs in linkage disequilibrium (LD) (*r^2^* ≥ 0.80) with the GWAS SNPs. Of the tested 1200 SNPs, 858 returned scores of 1–6 by RegulomeDB. Of these 858 SNPs, 97 were predicted to have regulatory functions with RegulomeDB score of < 3. Notably, only 8 of the 97 predicted regulatory variants were genome-wide significant SNPs (*LIPA*/rs2246833, RegulomeDB score = 1b; *ZC3HC1*/rs11556924, *CYP17A1-CNNM2-NT5C2*/rs12413409, *APOE-APOC1*/rs2075650 and *UBE2Z*/rs46522, each with a RegulomeDB score = 1f; *ZNF259-APOA5-APOA1*/rs964184, *SMG6*/rs2281727 and *COL4A1-COL4A2*/rs4773144, each with a RegulomeDB score = 2b). The remainder 89 functional SNPs were in linkage disequilibrium with GWAS SNPs. This study supports the hypothesis that some of the non-coding variants are true signals via regulation of gene expression at transcription level. Our study indicates that RegulomeDB is a useful database to examine the putative functions of large number of genetic variants and it may help to identify a true association among multiple tagged SNPs in a complex disease, such as CAD.

**Database URLs:**

http://www.regulomedb.org/; https://www.broadinstitute.org/mpg/snap/

## Background

Most human DNA sequence is non-coding (98%) and hence only small portion (2%) of human genome encodes proteins (1). Although the pathogenesis of monogenic disorders is largely explained, it has been difficult to determine the underlying mechanisms of complex disorders like coronary artery disease (CAD). Before the development of genome-wide association studies (GWAS), only the APOE*4 allele showed consistent association with the risk of CAD across many populations (2–5).

The hypothesis-free GWAS approach was designed with the assumption that common DNA variants explain the bulk of the variation in common diseases (6). About 90% of GWAS-implicated variants, exert only minimal to modest effect sizes on disease phenotypes, and they are present in non-coding rather than coding regions (7). Highly sensitive molecular and computational techniques have identified different regulatory elements (DNAse hypersensitive regions, sequences affecting the binding of transcription factors and promoters or enhancers) in intergenic regions (8). Common variants located in one of these regulatory elements may affect gene expression. To predict the role of these variants in gene regulation and to differentiate between physically tagged and functional single nucleotides polymorphism (SNPs), many databases have been created (9). RegulomeDB is one of such databases that describes the role of these variants in transcriptional regulation.

Similar to many other complex diseases, GWAS have identified hundreds of risk variants associated with CAD that need to be analyzed for their functional role in gene expression (10). Recently, we have used SNAP Webportal and Regulome DB to identify potential regulatory function of variants in associated risk loci for Alzheimer’s disease (11). In this study, we have applied the same approach to identify the regulatory nature of GWAS-implicated variants with CAD and those that are in linkage disequilibrium (LD) with these variants.

### Objective

The objective of our study was to assess the GWAS-implicated CAD variants and those variants in LD with GWAS variants for their potential regulatory effects on gene transcription using bioinformatics tools.

## Materials and methods

### SNPs selection

A total of 58 SNPs within 54 CAD loci was selected, including 52 with accepted genome-wide significant threshold (*P* < 5 × 10^−8^) and 6 with suggestive associations (*P* > 5 × 10^−8^) identified in two GWAS (12, 13). Detailed information on the selected 58 SNPs is provided in Supplementary Table S1.

### Linkage disequilibrium

For the LD assessment of the selected 58 SNPs, we used SNAP web portal (https://www.broadinstitute.org/mpg/snap/, accessed 13 July 2016) (14) (Supplementary Table S2). SNAP contains data from the Northern European from Utah (CEU) population derived from the 1000 Genomes Pilot Project 1 and three different releases of the International-Hap Map Project. We used data from both the 1000 Genomes Project and HapMap 3 (release 2) to identify SNPs in strong LD (*r^2^* ≥ 0.80) with our SNPs of interest. We did not select an array bound search, and query SNPs were included in the output. We performed the search at three thresholds—*r^2^* ≥ 0.80, *r^2^* ≥ 0.90 and *r^2^* ≥ 1.0—for both SNP datasets and identified a total of 1,200 SNPs in LD with the 58 published GWAS SNPs, including the GWAS SNPs themselves. As shown in Table 1, the number of proxy SNPs decreased with the increased level of *r^2^*.

**Table 1.** Number of SNPs in LD for all published GWAS SNPs for HapMap3 and 1000 genomes populations at tested *r^2^* threshold

		LD	
*r^2^*threshold	0.80	0.90	1.0
1000 Genomes	1176	928	480
Hap Map3	210	157	74
Total (overlaps removed)	1200	934	485

### Functional assessment of CAD-associated SNPs

We used RegulomeDB to identify potentially functional SNPs among the 1200 SNPs of interest. Regulome DB is a database that scores SNPs functionality based upon experimental data, such as its existence in a DNAase hypersensitive site or transcription factor binding site. These regions have been characterized biochemically, and data are drawn from published literature, Gene Expression Omnibus and ENCODE project that include a total of 962 experimental datasets, covering over 100 tissues and cell lines and representing nearly 60 million annotations. The output data can be mapped to Human genome version 19. It is a user friendly and freely accessible database (http://www.regulomedb.org/accessed 17 July 2016) (15). The functional Grades 1–6 of RegulomeDB are given in Table 2. SNPs showing the strongest evidence of being regulatory (affecting the binding of transcription factor) are given a score of 1 and SNPs demonstrating the least evidence of being functional are given a score of 6.

**Table 2.** RegulomeDB category summaries (15)

Category	Description
**Likely to affect binding and linked to expression of a gene target**	
1b	eQTL + TF binding + any motif + DNase footprint + DNase peak
1c	eQTL + TF binding + matched TF motif + DNase peak
1d	eQTL + TF binding + any motif + DNase peak
1e	eQTL + TF binding + matched TF motif
1f	eQTL + TF binding/DNase peak
**Likely to affect binding**	
2a	TF binding + matched TF motif + matched DNase footprint + DNase peak
2b	TF binding + any motif + DNase footprint + DNase peak
2c	TF binding + matched TF motif + DNase peak
**Less likely to affect binding**	
3a	TF binding + any motif + DNase peak
3b	TF binding + matched TF motif
**Minimal binding evidence**	
4	TF binding + DNase peak
5	TF binding or DNase peak
6	Motif hit

## Results

**Figure 1.**
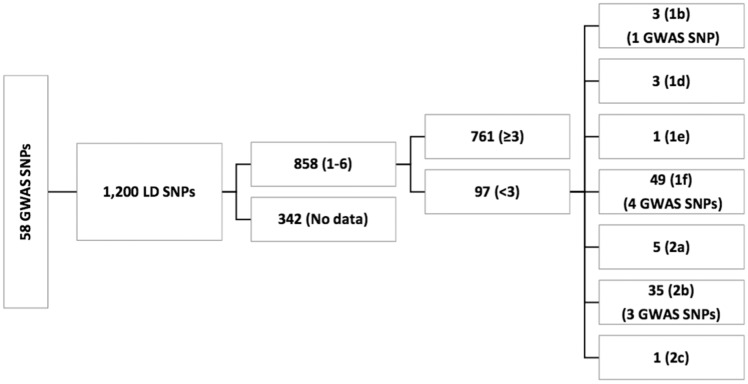
58 GWAS ANPs in LD with 1200 SNPs. We used SNAP webportal to determine LD SNPs. These 1200 SNPs were further evaluated by RegulomeDB to identify their functional role. RegulomeDB did not provide data for 342 SNPs. A total of 858 SNPs returned the scores of 1–6 by RegulomeDB. Of those 858 SNPs, 97 returned the scores of < 3. Among 97 functional SNPs, only 8 were GWAS SNPs. Lower the RegulomeDB score, more evidence of functionality.

Among the 1200 SNPs evaluated with RegulomeDB, 342 had no data (Supplementary Table S3). Of the 858 SNPs for which RegulomeDB provided a score, 97 had a score of <3 (likely to affect the binding) and among these only 8 SNPs were genome-wide significant, including *LIPA*/rs2246833 (RegulomeDB score = 1b; eQTL in monocytes), *ZC3HC1*/rs11556924 (RegulomeDB score = 1f; eQTL in monocytes), *CYP17A1-CNNM2-NT5C2*/rs12413409 (RegulomeDB score = 1f; eQTL in monocytes and lymphoblasts), *APOE-APOC1*/rs2075650, and *UBE2Z*/rs46522 (RegulomeDB score = 1f; eQTL in monocytes), *ZNF259-APOA5-APOA1*/rs964184, *UBE2Z*/rs46522, *SMG6*/rs2281727, *COL4A1-COL4A2*/rs4773144 (RegulomeDB score =2b; eQTLs in monocytes and lymphoblasts). A flow chart summarizes these results (Figure 1) . The remaining 89 SNPs with RegulomeDB scores < 3 were not identified in GWAS but they were in LD (*r^2^* ≥ 0.80) with the 29 GWAS reported SNPs. A summary of the regulatory SNPs in LD with GWAS SNPs is provided in Table 3.

**Table 3.** Functional SNPs (RegluomeDB Score < 3) in LD (*r^2^* ≥ 0.80) with published GWAS SNPs

GWAS SNPs	Functional proxy SNPs	Regulome DB score
*LIPA/rs2246833*	*LIPA/*rs1332327	2b
	*LIPA/*rs1332328	2b
	*LIPA/*rs1412444	1d
	***LIPA/*rs2246833**^a^	**1b**
	*LIPA/*rs2250644	2b
*ZC3HC1/*rs11556924	***ZC3HC1/*rs11556924**^a^	**1f**
*CYP17A1-CNNM2-NT5C2/*rs12413409	*AS3MT*/rs11191454	**1f**
	*BORCS7-ASMT/*rs4409766	1f
	*CNNM2/*rs10883808	1f
	*MAT2A/*rs1446668	2a
	*NT5C2/*rs10883832	1f
	*CNNM2/*rs11191479	1f
	*NT5C2/*rs11191557	1f
	*CNNM2/*rs11191499	1f
	*NT5C2/*rs11191558	1f
	*CNNM2/*rs11191514	1f
	*NT5C2/*rs11191580	1f
	*CNNM2/*rs11191515	1f
	*NT5C2/*rs1119158*2*	1f
	*CNNM2/*rs12221064	2b
	*NT5C2/*rs12412038	1f
	*CNNM2/*rs12411886	1f
	*NT5C2/*rs12413046	1f
	***CNNM2/*rs12413409**^a^	**1f**
	*NT5C2/*rs9633712	1e
	*CNNM2/*rs17115213	1f
	*NT5C2/*rs11191548	1f
	*CNNM2/*rs2297787	2a
	*CNNM2/*rs3781285	1f
	*CNNM2/*rs943037	1f
	*CNNM2/*rs12219901	2b
*APOE-APOC1/TOMM40*/rs2075650	***APOE-APOC1/*rs2075650**^a^	**1f**
*UBE2Z*/rs46522	*GIP/*rs2291725	1f
	*GIP/*rs4794004	1d
	*SNF8/*rs1994970	1f
	*SNF8/*rs4793992	1f
	*UBE2Z/*rs12601672	2b
	*UBE2Z/*rs15563	1f
	*UBE2Z/*rs3744608	2a
	*UBE2Z/*rs3848460	1f
	***UBE2Z/*rs46522**^a^	**1f**
	*UBE2Z/*rs11079844	1f
*ZNF259-APOA5-APOA1*/rs964184	***ZNF259-APOA5-APOA1/*rs964184**	1f
*SMG6*/rs2281727	***SMG6/*rs2281727**^a^	**2b**
	*SMG6/*rs7217687	2b
	*SMG6/*rs9908888	2b
*COL4A1-COL4A2*/rs4773144	***COL4A1-COL4A2/*rs4773144**^a^	**2b**
*ABO*/rs579459	*ABO/*rs649129	2b
		
*ADMTS7*/rs7173743	*LOC105370915/*rs5029904	2b
	*PHACTR1/*rs4773143	2b
*CXCL12*/rs501120	*CXCL12/*rs518594	2b
	*CXCL12/*rs1746052	2b
*FURIN-FES*/rs17514846	*FES/*rs1894401	1b
*HHIPL1/rs2895811*	*HHIPL1/*rs28391527	2b
	*HHIPL1/*rs4624107	2b
	*HHIPL1/*rs7145262	2b
*IL6R/rs4845625*	*IL6R/*rs7549250	2b
	*IL6R/*rs7549338	2b
	*IL6R/*rs7553796	2b
*KCNE2*/rs9982601	*KCNE2/*rs28591415	2b
*KIAA1462*/rs2505083	*KIAA1462/*rs3739998	2b
*LPL*/rs264	*LPL/*rs271	1f
	*LPL/*rs3779788	2b
*MIA3*/rs17465637	*MIA3/*rs17163301	2b
*PLG*/rs4252120	*PLG/*rs4252126	1f
	*PLG/*rs4252135	1f
*PPAP2B*/rs17114036	*LOC101929929/*rs72664304	2a
	*PLPP3/*rs4634932	1f
*SLC22A4-SLC22A5*/rs273909	*SLC22A5/*rs17689550	1f
	*SMG6/*rs7217687	2b
	*SMG6/*rs9908888	2b
*SORT1*/rs602633	*CELSR2/*rs12740374	2b
	*CELSR2/*rs629301	1f
	*CELSR2/*rs646776	1f
*TRIB1*/rs2954029	*LOC105375745/*rs2980853	2b
	*LOC105375745/*rs2001844	2b
	*LOC105375745/*rs6982636	2b
	*TRIB1/*rs2980856	2b
*VAMP5-VAMP8-GGCX*/rs1561198	*GGCX/*rs6738645	1f
	*GGCX/*rs10187424	1f
	*VAMP8/*rs1009	1b
	GGCX/rs6547621	1f
	*VAMP8/*rs1348818	1f
	*GGCX/*rs2886722	1f
	*VAMP8/*rs3770098	1f
	*VAMP8/*rs6757263	1f
*WDR12*/rs6725887	*ICA1L/*rs72934715	2b
	*NBEAL1/*rs2351524	1f
	*WDR12/*rs72936852	2b
	*NBEAL1/*rs4675310	1f
	*NBEAL1/*rs72934512	2b
*REST-NOA1/*rs17087335	*REST*/rs2227901	1f
	*REST-NOA1/*rs7687767	1d
*SWAP70*/rs10840293	*SWAP70/*rs93138	1f
	*SWAP70/*rs360136	1f
*SMAD3*/rs56062135	*SMAD3****/***rs17293632	2a
	*SMAD3****/***rs1866316	2b
	*MTERF1/*rs8032739	2b
*CDKN2BAS1*/rs1333049	*CDKN2BAS1*/rs4977574	2c

a GWAS significant SNPs with functional evidence (RegulomeDB score < 3) are bolded.

Overall, we had 97 functional SNPs (RegulomeDB < 3). Eight of these were GWAS SNPs, and the remaining 89 were in LD (*r*^2^ ≥ 0.80) with the GWAS SNPs.

Three variants, *FES*/rs1894401, *LIPA*/rs2246833 and *VAMP8*/rs1009, were strongly predicted to be functional with score of 1b. *FES/*rs1894401 is an intronic SNP that is an eQTL for *FES* in thyroid and transformed lymphoblasts, is present in the binding motif of Pax5, and affects the binding of eleven transcription factors. *LIPA*/rs2246833 (RegulomeDB score = 1b), located in Intron 6 of *LIPA*, in the DNA motif of EWSRCFLI1, is a GWAS reported SNP along with 4 other functional SNPs (of 12 tested) in this region and it and it affects the binding of CTCF. It is an eQTL in the whole blood. *VAMP5-VAMP8-GGCX*/rs1009 is in exon 3 of *VAMP8* and affects the binding of CTCF and HSF1. rs1009 of *VAMP8* is an eQTL in lymhoblasts, skeletal muscles, adipose tissue and thyroid. Of 42 SNPs analyzed in this locus, we found 8 other SNPs with RegulomeDB score < 3 (Table 3).

There were 33 functional SNPs within 15 GWAS identified CAD loci: *ABO* (1of 10 assessed), *ADAMTS7* (1 of 15 assessed), *CXCL12* (2 of 36 assessed), *HHIPL1* (3 of 17 assessed), *KCNE2*(2 of 18 assessed), *KIAA1462* (1 of 9 assessed), *MIA3* (1 of 27 assessed), *PPAP2B* (2 of 22 assessed), *SORT1* (3 of 9 assessed), *WDR12* (5 of 214 assessed), *IL6R* (3 of 14 assessed), *LPL* (2 of 6 assessed), *PLG* (2 of 41 assessed)*, SLC22A4-SLC22A5* (1 of 2 assessed) and *TRIB1* (4 of 16 assessed).

Of 97 SNPs with RegulomeDB score < 3, 25 were in the *CYP17A1-CNNM2-NT5C2* region, and one of them was a GWAS reported SNP (rs12413409). The regional LD plot of this SNP is given in Supplementary Figure S1. rs9633712 (RegulomeDB score = 1e) is located in Intron 3 of *NT5C2* and is an eQTL for *USMG5* in monocytes. This SNP was also found in the motifs of the following transcription factors: PU1, ELF1, Sfpil, PU.1 and c-Ets-1. It appears to affect the binding of SPI1. Twenty SNPs returned a score of 1f (likely to affect the binding), and 18 of them were in intronic regions. *NT5C2/*rs11191558 lies in HOXC series of DNA motifs, and *CNNM2*/rs3781285 lies between NF-kappaB and P50:50. *NT5C2*/rs2297787 returned a score of 2a, affecting the binding motifs of FOXI1, HNF3-alpha and FOXP1 and the binding of FOXA1. SNP rs12412038 is located in Intron 10 of *NT5C2* and is in the binding motif of Irx. The remaining two SNPs, rs12219901 and rs12221064, lie in the *CNNM2-NT5C2* intergenic region and upstream of *CNNM2*, respectively. They are located *in* DNA motifs of SRF and MAZR and affect the binding of POLRA2 and CTCF/ETS. Interestingly, rs943037 resides in exon 7 of *CNNM2*. Nineteen of the 25 SNPs in the region of *CYP17A1-CNNM2-NT5C2* are eQTLs for *USMG5* (Table 4).

**Table 4.** Putative functional SNPs and corresponding motifs, eQTL and related transcription factors (Regulome DB score < 3)

Coordinate 0-based	SNP ID	RegulomeDB score	Gene/Locus	Position	eQTL	Motif		Protein-binding
chr15:91429041	rs1894401	1b	*FES*	*Intron 2*	FES		Pax5	SPI1
								USF1
								POLR2A
								GABPA
								BHLHE40
								CEBPB
								CTCF
								MAX
								RFX5
								RUNX3
								STAT5A
chr10:91005853	rs2246833	1b	*LIPA*	*Intron 6*	LIPA		EWSR-FLI1	CTCF
							znf143	
chr2:85808736	rs1009	1b	*VAMP8*	*Exon 3*	VAMP8			CTCF
					LOC388969			HSF1
chr17:47038470	rs4794004	1d	*GIP*	*Intron 4*	ATP5G1		Gata5	NR3C1
					UBE2Z			IN3AK20
								CREB1
								TAF1
								TCF12
								CTCF
								POLR2A
								USF1
								FOXA1
								FOXA2
								RBBP5
chr10:91002926	rs1412444	1d	*LIPA*	*Intron3*	LIPA		SAP1a	ATF2
							ELK1	FOXM1
							ELK3	SP1
							ELK4	SPI1
							MECP2	MTA3
							ERF	RUNX3
							ERG	
							ETS1	
							ETV1	
							ETV2	
							ETV3	
							Gabpa	
chr10:104873760	rs9633712	1e	*NT5C2*	*Intron 3*	UMG5		PU1	SP11
							ELF-1	
							Sfpil	
							PU.1	
							c-Ets-1	
chr11:116648916	rs964184	1f	*ZPR1*	*Downstream ZRP1*	TAGLN		FOXJ2	
chr1:109818529	rs646776	1f	*CELSR2*	*Upstream CELSR2*	PSMA5			CTCF
								HEY1
								REST
								POLR2A
								ZBTB7A
								TAF7
chr10:104616662	rs4409766	1f	*BORCS7-ASMT*	*Intron 1*	C10orf77		Tcf3	BACH1
					USMG5			MAFF
								MAFK
chr17:47008206	rs4793992	1f	*SNF8*	*Intron 7*	ATP5G1			POLR2A
					UBE2Z			TEAD4
chr6:161152293	rs4252126	1f	*PLG*	*Intron 11*	PLG			CTCF
								RUNX3
								TEAD4
								RAD21
chr6:161154231	rs4252135	1f	*PLG*	*Intron 12*	PLG			CTCF
								FOXA1
								NFKB1
								RAD21
								ZNF263
								SMC3
								ZNF143
								FOXA2
chr10:104846177	rs11191548	1f	*NT5C2 gene region*	*Downstream NT5C2*	USMG5		TEAD1	
							TEAD3	
chr10:104864613	rs11191557	1f	*NT5C2*	*Intron 5*	USMG5			
chr10:104864677	rs11191558	1f	*NT5C2*	*Intron 5*	USMG5		HOXC13	
							Hoxa13	
							Hoxc13	
							Hoxd12	
							HOXA13	
							HOXD9	
							HOXC11	
chr10:104871203	rs12413046	1f	*NT5C2*	*Intron 3*	USMG5			NR3C1
								TRIM28
								CTCF
								ATF2
								IKZF1
								TCF7L2
								ZNF263
chr10:104871278	rs10883832	1f	*NT5C2*	*Intron 3*	USMG5			TRIM28
								TCF7L2
chr10:104913652	rs11191582	1f	*NT5C2*	*Intron 2*	USMG5			EP300
								NFIC
								TCF12
								TEAD4
								STAT1
								ARID3A
								EP300
								JUN
								RCOR1
chr10:104906210	rs11191580	1f	*NT5C2*	*Intron 2*	USMG5			TRIM28
								SETDB1
								GATA1
								GTF2F1
								CEBPB
								FOS
								JUND
								ZNF263
chr10:104856161	rs12412038	1f	*NT5C2*	*Intron 10*	USMG5		Irx-3	
							Irx-2	
							Irx-4	
							Irx-6	
chr2:85807081	rs1348818	1f	*VAMP8*	*Intron 2*	GGCX		HMGIY	EBF1,
							Mtf1	
							Srf	
							Zfp105	
							HMGIY	
chr2:85805366	rs3770098	1f	*VAMP8*	*Intron1*	VAMP8			POLR2A
					LOC388969			BHLHE40
								E2F6
								KDM5B
								MAX
								MXI1
								MYC
								NFIC
								WRNIP1
chr2:85803541	rs6757263	1f	*VAMP8*	*Upstream VAMP8*	GGCX			SP1
					VAMP8			EP300
					LOC388969			NFIC
chr17:46988596	rs46522	1f	*UBE2Z*	*Intron 2*	ATP5G1			NFKB
					UBE2Z			NFYB
								RUNX3
chr19:45395618	rs2075650	1f	*TOMM40*	*Intron 2*	TOMM40		RREB1	
chr1:56996190	rs4634932	1f	*PLPP3*	*Intron 2*	PPAP2B			POLR2A
chr2:203880833	rs4675310	1f	*NBEAL1*	*Intron 1*	ALS2CR13			
chr10:104681142	rs17115213	1f	*CNNM2*	*Intron 1*	USMG5			
chr10:104721125	rs10883808	1f	*CNNM2*	*Intron 1*	USMG5			
chr10:104723619	rs11191479	1f	*CNNM2*	*Intron 1*	USMG5			GATA1
								TAL1
								CEBPB
chr10:104773363	rs11191514	1f	*CNNM2*	*Intron 1*	USMG5			PAX5
chr10:104776526	rs11191515	1f	*CNNM2*	*Intron 1*	USMG5			
chr10:104825664	rs3781285	1f	*CNNM2*	*Intron 4*	USMG5		NF-kappaB	IKZF1
							P50:50	
chr10:104835918	rs943037	1f	*CNNM2*	*Exon 7*	USMG5		TBX20	
							Foxj1	
chr8:19813701	rs271	1f	*LPL*	*Intron 6*	LPL			
chr17:47039131	rs2291725	1f	*GIP*	*Exon 4*	GIP			GATA2
								TCF4
								FOSL2
								EGR1
								ELF1
								FOS
								NR3C1
								EP300
								RXRA
								CHD2
								JUND
								POLR2A
								RAD21
								FOSL1
								REST
chr2:203880991	rs2351524	1f	*NBEAL1*	*5' UTR*	ALS2CR13			
chr1:109818305	rs629301	1f	*CELSR2*	*3' UTR*	PSRC1			CTCF
								POLR2A
chr2:85774675	rs6547621	1f		*3' UTR*	GGCX			ELK4
								POLR2A
chr10:104660003	rs11191454	1f	*AS3MT*	*Intron10*	USMG5			
chr10:104685298	rs12411886	1f	*CNNM2*	*Intron1*	USMG5		Zec	
chr10:104719095	**rs12413409**	1f	*CNNM2*	*Intron1*	USMG5			POLR3A
chr10:104764270	rs11191499	1f	*CNNM2*	*Intron1*	USMG5			
chr17:47014126	rs1994970	1f	*SNF8*	*Intron4*	ATP5G1		TFII-I	
					UBE2Z			
chr2:85742296	rs2886722	1f	*Pseudogene*		LOC388969			TCF7L2
chr2:85783127	rs6738645	1f	*GGCX*	*Intron5*			Evi-1	POLR2A
chr2:85794296	rs10187424	1f	*Pseudogene*		GGCX			
					LOC388969			
chr5:131723064	rs17689550	1f			RAPGEF6			
chr7:129663495	rs11556924	1f	*ZC3HC1*	*Exon8*	KIAA0265			
chr17:4702833	rs11079844	1f	*Pseudogene*		ATP5G1			
chr17: 47005192	rs15563	1f	*UBE2Z*	*Exon7*	ATP5G1		PRDM1	
chr17:47047113	rs3848460	1f	*UBE2Z*		ATP5G1			CEBPB
chr10:104680136	rs2297787	2a	*CNNM2*	*Intron 1*			Freac-7	FOXA1
							HFH3(FOXI1)	SIN3A
							HNF3alpha	ZNF263
							FOXP1	HNF4G
							Elf3	FOXA1
							Foxl1	
							Srf	
							Tcf3	
							Tcfap2e	
							Zfp105	
							HFH(FOXl1)	
chr1:56948289	rs72664304	2a	*C8B*	*Intron 6*			FOXA1	FOXA1
							Foxa2	FOXA2
								TCF4
								SP1
								HNF4G
								HNF4A
								HDAC2
								JUND
								EP300
chr17:46993232	rs3744608	2a	*UBE2Z*	*Intron 3*			Zfp740	SPI1
							MZF1	POLR2A
							MAZR	IKZF1
							SP1	MAX
							SP1:SP3	TFAP2A
							WT1ZNF21	TFAP2C
							Zfp281	SP1
							ZFp740	CEBPB
							WT1	NR3C1
							ZNF219	BATF
							ZNF740	BCL11A
							SP4	MEF2A
								NFKB1
								JUND
								EP300
								STAT3
								IRF4
								EBF1
								FOSL2
								BATF
								NR3C1
								RUNX3
								MYC
								STAT3
chr2:85764959	rs1446668	2a	*MAT2A*	*nc transcript*			CTCF	CTCF
				*Upstream MAT2A*				POLR2A
								TAF1
								RFX5
								RAD21
								HEY1
								CDX2
								HNF4A
								ZNF263
								NR3C1
								CTCF
								MYC
								AR
								MYBL2
								TEAD4
								MAZ
								CHD2
								SMC3
								TBP
								ZNF143
								CDX2
								E2F6
								MAX
								NR3C1
								SIN3A
								YY1
								REST
								HMGN3
chr17:47006492	rs12601672	2b	*UBE2Z*	*Downstream UBE2Z*			Zfx	POLR2A
								EGR1
								SPI1
								ELF1
chr10:30316071	rs3739998	2b	*KIAA1462*	*Exon 2*			RELA	CTCF
								MYC
								PAX5
								ZNF143
chr8:126476378	rs2980856	2b	*TRIB1 gene*	*Intergenic region*			pax-8	JUND
			*region*	*Downstream TRIB1*			Sox17	POLR2A
								TFAP2C
								MXI1
								CEBPB
chr9:136154303	rs649129	2b	*ABO gene region*	*Intergenic region*			IRF	NFYA
				*Upstream ABO*				POLR2A
								FOS
								IRF1
								NFYB
								PML
chr10:104840966	rs12219901	2b	*CNNM2 gene*	*Intergenic region*			SRF	POLR2A
			*region*	*Downstream CXCL12*				
chr10:44778545	rs1746052	2b	*CXCL12 gene*	*Intergenic region*			GATA1	TAL1
			*region*	*Downstream CXCL12*				
chr21:35593826	rs28451064	2b	*LINC00310 gene*	*Intergenic region*			PPAR	SP1
			*region*	*Downstream*				FOXA2
				*LINC00310*				
chr17:2098271	rs7217687	2b	*SMG6*	*Intron 13*	-		NF-1	SIN3A
								TCF12
								MAX
								YY1
								ZNF263
								EP300
								TEAD4
chr13:110960711	rs4773144	2b	*COL4A2*	*Intron 3*			STAT3:STAT3	POLR2A
								EZH2
chr14:100116251	rs28391527	2b	*HHIPL1*	*Intron 3*			MyoD	BHLHE40
							SCRT1	USF1
							FIGLA	FOXA1
								MAX
chr1:154404335	rs7549250	2b	*IL6R*	*Intron 3*			TBX15	MXI1
								FOS
								JUNB
								MAX
								JUND
								JUN
								STAT3
								FOSL1
								MAFK
								RCOR1
								MYC
								USF2
								TEAD4
								RCOR1
								YY1
chr1:154404379	rs7549338	2b	*IL6R*	*Intron 3*			GR	FOS
							AR	JUNB
								JUND
								JUN
								STAT3
chr1:154404405	rs7553796	2b	*IL6R*	*Intron 3*			NF-kappaB,	FOS
								JUND
								JUN
								STAT3
chr14:100127439	rs4624107	2b	*HHIPL1*	*Intron 7*			Pax5	JUND
chr10:91011457	rs1332328	2b	*LIPA*	*Intron 9*			UF1H3BETA	CREBBP
								ZNF263
								CDX2
								ELF1
								ZEB1
								TBP
								TFAPC2
								TBP
								POLR2A
								ETS1
								GABPA
								HEY1
chr17:2117944	rs2281727	2b	*SMG6*	*Intron 13*			SRY	CREBBP
							Srf	EP300
							Zfp105	STAT3
								TRIM28
								MYC
								RBBP5
chr8:126479314	rs6982636	2b	*LOC105375745*	*Intron1*			MAF	SMARCC1
								RFX3
								POLR2A
								GATA2
								CHD2
								GTF2F1
chr14:100125720	rs7145262	2b	*HHIPL1*	*Intron4*			ESR2	SMARC4
								ZBTB7A
								SMARB1
								POLR2A
								EZH2
								RAD21
								BACH1
chr10:104677125	rs12221064	2b	*CNNM2*	*Upstream CNNM2*			MAZR,	CTCF, ETS1
								ETS1
chr8:126478349	rs2980853	2b	*LOC105375745*	*Upstream*			Pit-1,	RFX3
				*LOC105375745*				
chr15:79152421	rs5029904	2b	*LOC105370915*	*Upstream*			NeuroD	USF1
				*LOC105370915*				POLR2A
								YY1
								FOXA1
								E2F4
								MAX
								TAF7
								TAF1
								MXI1
chr8:126478744	rs2001844	2b	*LOC105375745*	*Upstream*			HSF1	RFX3
				*LOC105375745*				
chr10:91011680	rs1332327	2b	*LIPA*	*5' UTR*			AP-4,	CREBBP
								CDX2
								ELF1
								TBP
								SPI1
								NRF1
								ELF1
								ETS1
								GABPA
								SPI1
								PAX5
								SREBF1
chr17:2102452	rs9908888	2b	*SMG6*	*Intron10*			GR	CEBPB
							AR	
chr10:91008878	rs2250644	2b	*LIPA*	*Intron1*			Oct-1	RUNX3
							XBP-1	
							MAfb	
							Mafk	
							MAFB	
							MAFK	
							NRL	
chr1:109817589	rs12740374	2b	*CELSR2*	*Exon34*			HNF1	EBF1
							HNF1A	
							DUXA	
chr1:222794090	rs17163301	2b	*MIA3*	*Intron1*			HNF1	EBF1
							HNF1A	
							HNF1B	
							DUXA	
chr21:35644028	rs28591415	2b	*pseudogene*				PPAR	EP300
								FOXA1
								HDAC2
								NFIC
								SP1
chr2:203713279	rs72934715	2b	*ICA1L*	*Intron2*			HMGIY	ATF2
								NFIC
								EBF1
								EP300
								NFKB1
								PAX5
chr2:203775474	rs72936852	2b	*WDR12*	*Intron1*			AR	MAFF
chr2:203926270	rs72934512	2b	*NBEAL1*	*Intron6*			TEAD1	TEAD4
							TEAD3	
chr8:19803092	rs3779788	2b	*LPL*	*Intron1*			TGIF	CEBPB
chr10:44757106	rs518594	2b	*CXCL12*	*Downstream intergenic*			E2A	FOXM1
							NRSE	NFIC
							NRSF	MAX
								EBF1
								TBL1XR1
chr4:57824931	rs7687767	1d	*CECR6*				Sox8	FOXA1
chr11:5759712	rs93138	1f	*SWAP70*	*Intron8*				
	rs360136	1f	*SWAP70*	*Exon13*				
chr:457798188	rs2227901	1f	*REST-NOA1*	*Exon6*				SPI1
chr:1567442595	rs17293632	2a	*SMAD3*	*Intron4*			Bach1	SIN3A
							AP-1	TCF7L2
							JundM2	TFAP2A
							Pou1f1	TFAP2C
							Pou3f1	ZNF217
							Sox5	POLR2A
							JDP2	STAT1
								MXI1
								TAF1
								E2F1
								POLR2A
								HDAC1
								TCF7L2
								MYC
								POLR2A
								ESR1
								EP300
								CDX2
								HNF4A
								CEBPB
								EP300
								FOS
								FOSL1
								GATA3
								JUNB
								JUN
								MYC
								NR2F2
								NR3C1
								RAD21
								RCOR1
								TCF12
								JUND
								MAFK
								EGR1
								MAX
chr15:67441996	rs18866316	2b	*SMAD3*	*Exon4*			AIRE	ESR1
							Elf3	NR3C1
							Srf	POLR2A
							Tcf3	
							Tcfap2e	
chr15:67448898	rs8032739	2b	*MTERF*	*Intron4*			SRF	CEBPB
								FOS
								MYC
								STAT3
chr9:22098573	rs4977574	2c	*CDKN2BAS1*	*Intron16*			AR	AR
							NR3C1	
							NR3C2	
							Ar	
							Elk-1	
							c-Ets-1(p54)	

One SNP rs2075650 lies in Intron 2 of *ApoEApoC1/TOMM40* with a RegulomeDB score of 1f. It is located in RREB1 DNA motif and is an eQTL for *TOMM40* (Table 4).

In total 3 of 107 SMG6 associated SNPs, rs2281727, rs7217687 and rs9908888 had a score of 2b and they affect the binding of EP300. rs2281727 is a genome-wide significant SNP located in Intron 9 of *SMG6*. It is in binding motifs of SRY, Srf and Zfp105 and affects the binding of CREBBP, EP300, STAT3, TRIM28, MYC and RBBP5 (Table 4).

The *UBE2Z* region had 10 functional SNPs, including a GWAS reported SNP, *UBE2Z*/rs46522 (RegulomeDB score of 1f). The SNP with the most evidence of regulatory function in this locus is rs4794004 with a score of 1d. It is in DNA motif of Gata5 that alters the expression of *UBE2Z* and *ATP5G1*and affects the binding of NR3C1, IN3AK20, CREB1, TAF12, CTCF, POLR2A, USF1, FOXA1, FOXA2 and RBBP5. The other 5 SNPs in this region have a score of 1f. The remaining two regulatory SNPs, rs3744608 and rs12601672, have scores of 2a and 2b, respectively. rs3744608 is located in Intron 3 of *UBE2Z* and it affects the binding of large number of transcription factors (Table 4).

*COL4A1-COL4A2*/rs473144 is a GWAS reported SNP, achieving a RegulomeDB score of 2b. This SNP lies in Intron 3 of *COL4A2* between STST3:STAT3 DNA motif and affects the binding of POLR2A and EZH2 (Table 4). *ZNF259-APOA5-APOA1*/rs964184 is a GWAS significant SNP with a score of 1f and is an eQTL for TAGLN. This SNP is located downstream of this gene region and is present in FOXJ2 DNA motif. Another GWAS significant SNP, *ZC3HC1*/rs11556924 is an exonic variant and the only functional SNP (score = 1f) in this locus; it is also an eQTL for *ZC3HC1* (Table 4).

*REST-NOA1/*rs17087335 is in LD with two functional SNPs (rs2227901 and rs7687767 with RegulomeDB scores of 1f and 1d, respectively). rs768776 lies in DNA motif of Sox8 and affects the binding of FOXA1. *SWAP70* has two functional SNPs, rs93138 and rs360136, each with a RegulomeDB score of 1f. *SWAP70*/rs93138 is an eQTL as evidenced in monocytes.

*SMAD3* has three functional SNPs, *SMAD3****/***rs17293632 and *SMAD3****/***rs1866316 and *MTERF1/*rs8032739 with RegulomeDB scores of 2a, 2a and 2b, respectively. Both are in LD with a lead GWAS SNP (*SMAD3*/rs56062135). *CDKN2BAS1/*rs1333049 has one functional SNP(rs4977574) only with RegulomeDB score of 3c. It is a part of a gene cluster on chromosome 9p21 and it maps to Intron 16 of cyclin dependent kinase, an important regulator of cell cycle.

## Discussion

Following the sequencing of human genome, a large number of SNPs have been identified that affect disease phenotypes, but their exact roles remain unclear (16). One possible explanation is that some variation affects disease expression at the transcriptional level other than at the protein level. For example, a base pair change in a transcription factor binding site may affect the binding affinity of transcription factors that consequently may alter the transcription of the related genes. These effects are indirect and may seem subtle, but their interactions with other genetic or environmental factors may result in the pathogenesis of common diseases.

Like other complex disorders, a large number of CAD associated risk variants have been discovered by multiple GWAS (12, 13, 17). ENCODE provides information regarding the functionality of human genome (18). This data requires careful interpretation and helps to define the biological function of previously termed ‘junk DNA’. Using bioinformatics tools, we may generate new hypotheses about the gene regulation of complex disorders. In this study, we have used two bioinformatics tools, SNAP and RegulomeDB, in order to identify the putative roles of CAD-associated SNPs.

We examined a total 1,200 SNPs in 54 loci implicated by GWAS, including 58 genome-wide significant SNPs. Ninety-seven SNPs were predicted to have regulatory functions with a RegulomeDB score of <3, but only 8 of them were genome-wide significant. Interestingly, all 8 genome-wide significant SNPs with suggested regulatory function are located either in intronic or intergenic regions, suggesting that these are true associations that regulate gene expression at the transcriptional level.

Among these eight GWAS reported functional SNPs, the SNP with the top RegulomeDB score was *LIPA*/rs2246833 (Regulome DB score = 1b). This variant is located in Intron 6 of lipase A (*LIPA*) and is an eQTL for the same gene which catalyzes intracellular triglyceride and hydrolyses cholesterol ester (19).

*ZC3HC1/*rs11556924 is a GWAS significant CAD associated SNP that returned a score of 1f. rs11556924 is a coding SNP located in the *ZC3HC1* gene region encoding NIPA (Nuclear Interaction Partner of ALK) protein. This polymorphism is responsible for arginine-histidine amino acid alteration at position 363 (R363H). The SNP has been associated with essential hypertension in Finnish population (20, 21).

The *CYP17A1-CNNM2-NT5C2* gene region has the highest number of regulatory SNPs, including one GWAS significant SNP, rs12413409. This locus affects diastolic blood pressure, systolic blood pressure and body mass index. All three measures are important risk factors for CAD (22). There are 25 putative regulatory SNPs in LD with rs12413409 that are located across four genes on chromosome 10 (*CNNM2, NT5C2, AS3MT* and *BORCS7/ASMT*) but affect the expression of same protein USMG5. These findings suggest that USMG5 should be investigated as an important player for CAD pathogenesis. USMG5 (upregulated during skeletal muscle growth protein 5) is also known as diabetes-associated protein in insulin-sensitive tissues that plays a crucial role in the maintenance of ATP synthase structure in mitochondria (23). Chen et al. (24) have purified this protein from bovine heart mitochondria and suggested its role in cell energy metabolism.

*APOE-APOC1/TOMM40*/rs2075650 is present in the *TOMM40* gene region near the *APOE-C1* cluster. *TOMM40* encodes TOMM40 protein, which is an important subunit 40 of outer mitochondrial membrane protein complex. rs2075650 risk allele has shown an association with low levels of CRP in CAD patients (25).

rs46522, an intronic SNP in Ubiquitin-conjugating enzyme E2Z (*UBE2Z*) gene region returns a RegulomeDB score of 1f. This SNP is associated with CAD in Iranian and Han Chinese populations (26, 27). The exact mechanism by which genetic alteration in *UBE2Z* can attribute to the CAD risk is not yet clear; however, rs46522 is in strong LD with the causal SNPs in gastric inhibitory peptide (GIP) gene that encodes GIP protein, a protein that modifies the glucose and lipid metabolism potentially mediating known CAD risk factors.

*ZNF259-APOA5APOA1*/rs964184 is also an important regulatory SNP. ZNF259 protein polymorphism has been associated with metabolic syndrome in Chinese population. Aung et al. (28) have also shown its association with lipid levels. *ZNF259* is located close to *APOA5*. Overexpression of *APOA5* in mice reduces plasma triglyceride levels and mice lacking *APOA5* have hypertriglyceridemia (29).

*COL4A2*/rs4773144 has been identified as functional lead SNP by RegulomeDB (score = 2b). This gene controls collagen proliferation, indicating a potential functional role in atherosclerotic plaque strengthening (30).

*SMG6*/rs2281727 is an intronic SNP. The potential function of SMG6 in CAD is not yet established. This gene promotes the endonuclease activity and is responsible for protection of telomere ends of chromosomes (16).

Although regulatory elements are most often found in non-coding regions of the genome, we found 5 loci with exonic regulatory SNPs (*VAMP8*/rs1009, *CNNM2*/rs943037, *GIP*/rs2291725, *KIAA1462*/rs3739998 and *UBE2Z*/rs15563), indicating the presence of regulatory signals inside the coding sequences as well.

USF1 is an upstream transcription factor whose binding is affected by three SNPs (*GIP*/rs4794004, *FES*/rs1894401, *HHIPL1*/rs28391527), suggests a potential functional link between *FES, GIP* and *HHIPL1* (31).

RegulomeDB identified three important functional SNPs affecting CAD phenotype. Among these, *REST-NOA1/*rs17087335 is the lead GWAS SNP that encodes a transcription factor which suppresses the voltage gated sodium and potassium channels and it has shown to maintain vascular smooth muscle cells in non-proliferative phase (32). *SWAP70*/rs10840293 encodes a signaling molecule that is implicated in cell adhesion and migration and it appears to be a potential regulator of leukocyte migration and their adhesion to endothelial cells (33). *SMAD3* is a major regulator of TGF-ß. A study on mice has shown that mutations in this gene lead to decreased connective tissue deposition in response to vascular injury (34).

It should be noted that 342 SNPs had returned ‘No Data’ when queried by RegulomeDB. This suggests that current evidence does not support a functional role for those variants. Our results also showed that some loci harbor markedly more regulatory SNPs as compared with other regions. We caution against interpreting this finding to meant that one region is more functionally relevant, as regions with ‘fewer’ functional SNPs may have yet to be interrogated as thoroughly and thus have fewer annotations.

Since these loci are mostly in Europeans, and only 5 of them are replicated in South Asians (35), the findings may not be as relevant to other populations as they are to Europeans as genetic effects can differ across populations. The cause of this varying association with disease phenotype may be the ethnic admixture resulting in population stratification. It is also noteworthy that robust associations of variants with different diseases have been reported in Europeans while other populations (Africans, Asians and Hispanics) failed to demonstrate those associations (36, 37).

Though the cellular mechanisms underlying CAD pathogenesis are established, the molecular basis is not yet agreed upon. Comprehending the molecular basis of disease is crucial before pathogenesis is completely described. The study has identified 97 regulatory SNPs associated with CAD. In summary, our results highlight the importance of considering both disease-associated SNPs and those SNPs in LD, as well as the regulatory function of these SNPs to help identify the causal genetic mechanisms of CAD. The methods which we have implemented here can inform planning of more complete and better directed functional genomic studies.

## Supplementary data

Supplementary data are available at *Database* Online.

## Funding

This study was partially supported by Higher Education Commission of Pakistan and the US National Institutes of Health grants (AG030653 and AG041718).

*Conflict of interest*. None declared.

## Supplementary Material

Supplementary Table 1

Supplementary Table 2

Supplementary Table 3

Supplementary Figure 1
