# Supplementary material for: Proficiency of data interpretation: identification of signaling SNPs/specific loci for coronary artery disease
Source: Database (Oxford). 2017 Oct 31;2017:bax078. doi: 10.1093/database/bax078 (PMC5737196; doi:10.1093/database/bax078)
Supplement: Supplementary Table 1 [file bax078_supp_table_s1.docx]

**Table S1: Coronary artery disease (CAD) GWAS variants**

**subjected to SNAP tool and RegulomeDB**

| ***GENE*** | **Ch #** | **SNP** | **GWAS p-value** | **Ethnicity** | **Reference** |
| --- | --- | --- | --- | --- | --- |
| *SORT1* | 1 | rs602633 | 1.47 x 10-25 | Europeans | 12 |
| *PCSK9* | 1 | rs11206510 | 1.79x10-05 | Europeans | 12 |
| *PPAP2B* | 1 | rs17114036 | 5.80 × 10–12 | Europeans | 12 |
| *MIA3* | 1 | rs17465637 | 6.06 × 10−05 | Europeans | 12 |
| *IL6R* | 1 | rs4845625 | 3.64 × 10–10 | Europeans | 12 |
| *WDR12* | 2 | rs6725887 | 1.16 × 10–15 | Europeans | 12 |
| *APOB* | 2 | rs515135 | 2.56 × 10–10 | Europeans | 12 |
| *ZEB2-AC074093.1* | 2 | rs2252641 | 5.30 × 10–8 | Europeans | 12 |
| *VAMP5-VAMP8-GGCX* | 2 | rs1561198 | 1.22 × 10–10 | Europeans | 12 |
| *ABCG5-ABCG8* | 2 | rs46522 | 2.12 × 10–9 | Europeans | 12 |
| *MRAS* | 3 | rs9818870 | 2.62 × 10–9 | Europeans | 12 |
| *GUCY1A3* | 4 | rs7692387 | 2.65 × 10–11 | Europeans | 12 |
| *EDNRA* | 4 | rs1878406 | 2.54 × 10–8 | Europeans | 12 |
| *REST-NOA1* | 4 | rs17087335 | 7.67×10-08 | All | 13 |
| *SLC22A4-SLC22A5* | 5 | rs273909 | 9.62 × 10–10 | Europeans | 12 |
| *TCF21* | 6 | rs12190287 | 4.94 × 10–13 | Europeans | 12 |
| *SLC22A3-LPAL2-LPA* | 6 | rs2048327 | 4.90 × 10–05 | Europeans | 12 |
| *KCNK5* | 6 | rs10947789 | 9.81 × 10–9 | Europeans | 12 |
| *PLG* | 6 | rs4252120 | 4.88 × 10–10 | Europeans | 12 |
| *ANKS1A* | 6 | rs17609940 | 1.36 × 10–8 | Europeans | 12 |
| *PHACTR1* | 6 | rs9369640 | 7.53 × 10–22 | Europeans | 12 |
| *ZC3HC1* | 7 | rs11556924 | 6.74 × 10–17 | Europeans | 12 |
| *HDAC9* | 7 | rs2023938 | 4.94 × 10–8 | Europeans | 12 |
| *KIAA1462* | 7 | rs10953541 | 3.12x10-8 | Europeans | 12 |
| *NOS3* | 7 | rs3918226 | 1.70×10-09 | All | 13 |
| *LPL* | 8 | rs264 | 2.88 × 10–9 | Europeans | 12 |
| *TRIB1* | 8 | rs2954029 | 4.75 × 10–9 | Europeans | 12 |
| *CDKN2BAS1* | 9 | rs3217992 | 7.75 × 10–57 | Europeans | 12 |
| *ABO* | 9 | rs579459 | 2.66 × 10–8 | Europeans | 12 |
| *CYP17A1-CNNM2-NT5C2* | 10 | rs12413409 | 6.26 × 10–8 | Europeans | 12 |
| *KIAA1462* | 10 | rs2505083 | 1.35 × 10–11 | Europeans | 12 |
| *CXCL12* | 10 | rs501120 | 1.79 × 10–9 | Europeans | 12 |
| *CXCL12* | 10 | rs2047009 | 1.59 × 10–9 | Europeans | 12 |
| *LIPA* | 10 | rs2246833 | 9.49× 10–06 | Europeans | 12 |
| *PDGFD* | 11 | rs974819 | 3.55 × 10–11 | Europeans | 12 |
| *ZNF259-APOA5-APOA1* | 11 | rs964184 | 1.02 × 10–17 | Europeans | 12 |
| *SWAP70* | 11 | rs10840293 | 1.30×10-08 | All | 13 |
| *SH2B3* | 12 | rs3184504 | 5.44 × 10–11 | Europeans | 12 |
| *KSR2* | 12 | rs11830157 | 3.90×10-04 | All | 13 |
| *COL4A1-COL4A2* | 13 | rs4773144 | 1.43 × 10–11 | Europeans | 12 |
| *COL4A1-COL4A2* | 13 | rs9515203 | 5.85 × 10–12 | Europeans | 12 |
| *FLT1* | 13 | rs9319428 | 7.32 × 10–11 | Europeans | 12 |
| *HHIPL1* | 14 | rs2895811 | 4.08 × 10–10 | Europeans | 12 |
| *ADAMTS7* | 15 | rs7173743 | 6.74 × 10–13 | Europeans | 12 |
| *FURIN-FES* | 15 | rs17514846 | 9.33 × 10–11 | Europeans | 12 |
| *SMAD3* | 15 | rs56062135 | 4.50×10-09 | All | 13 |
| *MFGE8-ABHD2* | 15 | rs8042271 | 3.70×10-08 | All | 13 |
| *RAI1-PEMT-RASD1* | 17 | rs12936587 | 1.24 × 10–09 | Europeans | 12 |
| *UBE2Z* | 17 | rs46522 | 1.81 × 10–08 | Europeans | 12 |
| *SMG6* | 17 | rs2281727 | 7.83 × 10–09 | Europeans | 12 |
| *BCAS3* | 17 | rs7212798 | 1.90×10-08 | All | 13 |
| *PMAIPI-MC4R* | 18 | rs663129 | 3.10×10-08 | All | 13 |
| *APOE-ApoC1* | 19 | rs2075650 | 5.86 × 10-11 | Europeans | 12 |
| *ApoE-ApoC1* | 19 | rs445925 | 8.76 × 10–09 | Europeans | 12 |
| *LDLR* | 19 | rs1122608 | 6.33 × 10–14 | Europeans | 12 |
| *ZNF507-LOC400684* | 19 | rs12976411 | 9.10×10-03 | All | 13 |
| *KCNE2* | 21 | rs9982601 | 7.67 × 10–17 | Europeans | 12 |
| *POM121L9P* | 22 | rs180803 | 1.60×10-10 | All | 13 |
