# Supplementary material for: Proficiency of data interpretation: identification of signaling SNPs/specific loci for coronary artery disease
Source: Database (Oxford). 2017 Oct 31;2017:bax078. doi: 10.1093/database/bax078 (PMC5737196; doi:10.1093/database/bax078)
Supplement: Supplementary Table 2 [file bax078_supp_table_s2.docx]

**Table S2: Putative functional SNPs (RegulomeDB score < 3) in linkage disequilibrium (*r^2^* ≥0.80) with GWAS significant SNPs**

| **Gene/SNP** | ≥0.80 | ≥0.90 | =1.0 |
| --- | --- | --- | --- |
| ***KCN5*/rs10947789^a,b^** | rs10947789 | rs10947789 | rs10947789 |
|  | rs10947787 | rs10947787 | rs10947787 |
|  | rs55818834 | rs55818834 | rs55818834 |
|  | rs4711585 | rs4711585 | rs4711585 |
|  | rs12200013 | rs12200013 | rs12200013 |
|  | rs12190363 | rs12190363 | rs12190363 |
|  | rs12207689 | rs12207689 | rs12207689 |
|  | rs12202900 |  |  |
|  | rs4714223 |  |  |
|  | rs6918122 |  |  |
|  | rs2033226 |  |  |
|  | rs10947788 |  |  |
|  |  |  |  |
| ***KIAA1462/*rs10953541^a,b^** | rs10953541 | rs10953541 | rs10953541 |
|  | rs7785962 | rs7785962 | rs7785962 |
|  | rs35024078 | rs35024078 | rs35024078 |
|  | rs34273277 | rs34273277 | rs34273277 |
|  | rs35061190 | rs35061190 | rs35061190 |
|  | rs35531458 | rs35531458 | rs35531458 |
|  | rs68170813 | rs68170813 | rs68170813 |
|  | rs13234124 | rs13234124 |  |
|  | rs71566736 | rs71566736 |  |
|  | rs34523267 |  |  |
|  |  |  |  |
| ***PCSK9*/rs11206510** | rs11206510 | rs11206510 | rs11206510 |
|  |  |  |  |
| ***LDLR*/rs1122608** | rs1122608 | rs1122608 | rs1122608 |
|  | rs112369586 | rs112369586 | rs112369586 |
|  | rs3786722 | rs3786722 | rs3786722 |
|  | rs55948246 | rs55948246 | rs55948246 |
|  | rs12052058 | rs12052058 | rs12052058 |
|  | rs3786728 | rs3786728 | rs3786728 |
|  | rs12052201 | rs12052201 | rs12052201 |
|  | rs73013202 | rs73013202 | rs73013202 |
|  | rs12609863 | rs12609863 |  |
|  | rs73013198 | rs73013198 |  |
|  | rs73015007 | rs73015007 |  |
|  | rs3786723 |  |  |
|  |  |  |  |
| ***ZC3HC1/*rs11556924** | rs11556924 | rs11556924 | rs11556924 |
|  |  |  |  |
| ***TCF21*/rs12190287** | rs12190287 | rs12190287 | rs12190287 |
|  | rs162185 |  |  |
|  |  |  |  |
| ***CYP17A1-CNNM2-NT5C2/rs12413409*** | rs12413409 | rs12413409 | rs12413409 |
|  | rs10883808 | rs10883808 | rs10883808 |
|  | rs78260931 | rs78260931 | rs78260931 |
|  | rs11191479 | rs11191479 | rs11191479 |
|  | rs10883806 | rs10883806 | rs10883806 |
|  | rs11191472 | rs11191472 | rs11191472 |
|  | rs10883815 | rs10883815 | rs10883815 |
|  | rs5011520 | rs5011520 | rs5011520 |
|  | rs112390216 | rs112390216 | rs112390216 |
|  | rs77860422 | rs77860422 | rs77860422 |
|  | rs10509759 | rs10509759 | rs10509759 |
|  | rs12411886 | rs12411886 | rs12411886 |
|  | rs78821730 | rs78821730 | rs78821730 |
|  | rs77602510 | rs77602510 | rs77602510 |
|  | rs17115213 | rs17115213 | rs17115213 |
|  | rs2297787 | rs2297787 | rs2297787 |
|  | rs12221064 | rs12221064 | rs12221064 |
|  | rs11191499 | rs11191499 | rs11191499 |
|  | rs11191502 | rs11191502 | rs11191502 |
|  | rs11191505 | rs11191505 | rs11191505 |
|  | rs74444347 | rs74444347 | rs74444347 |
|  | rs12221335 | rs12221335 | rs12221335 |
|  | rs77180047 | rs77180047 | rs77180047 |
|  | rs12221193 | rs12221193 | rs12221193 |
|  | rs11191514 | rs11191514 | rs11191514 |
|  | rs77787671 | rs77787671 | rs77787671 |
|  | rs11191515 | rs11191515 | rs11191515 |
|  | rs11191454 | rs11191454 | rs11191454 |
|  | rs11191453 | rs11191453 | rs11191453 |
|  | rs35125602 | rs35125602 | rs35125602 |
|  | rs74233296 | rs74233296 | rs74233296 |
|  | rs11191519 | rs11191519 | rs11191519 |
|  | rs75970938 | rs75970938 | rs75970938 |
|  | rs79668541 | rs79668541 | rs79668541 |
|  | rs76752100 | rs76752100 | rs76752100 |
|  | rs3740390 | rs3740390 | rs3740390 |
|  | rs11191434 | rs11191434 | rs11191434 |
|  | rs11191531 | rs11191531 | rs11191531 |
|  | rs12219027 | rs12219027 | rs12219027 |
|  | rs11191543 | rs11191543 | rs11191543 |
|  | rs3781285 | rs3781285 | rs3781285 |
|  | rs943037 | rs943037 | rs943037 |
|  | rs12219901 | rs12219901 | rs12219901 |
|  | rs10458729 | rs10458729 | rs10458729 |
|  | rs11191548 | rs11191548 | rs11191548 |
|  | rs11191551 | rs11191551 | rs11191551 |
|  | rs17094683 | rs17094683 | rs17094683 |
|  | rs12217501 | rs12217501 | rs12217501 |
|  | rs12220743 | rs12220743 | rs12220743 |
|  | rs12412038 | rs12412038 | rs12412038 |
|  | rs11191555 | rs11191555 | rs11191555 |
|  | rs11191557 | rs11191557 | rs11191557 |
|  | rs11191558 | rs11191558 | rs11191558 |
|  | rs11191560 | rs11191560 | rs11191560 |
|  | rs12413046 | rs12413046 | rs12413046 |
|  | rs10883832 | rs10883832 | rs10883832 |
|  | rs77055135 | rs77055135 | rs77055135 |
|  | rs9633712 | rs9633712 | rs9633712 |
|  | rs79082900 | rs79082900 | rs79082900 |
|  | rs1060240 | rs1060240 | rs1060240 |
|  | rs10883835 | rs10883835 | rs10883835 |
|  | rs732998 | rs732998 | rs732998 |
|  | rs11191575 | rs11191575 | rs11191575 |
|  | rs79993475 | rs79993475 | rs79993475 |
|  | rs12220375 | rs12220375 | rs12220375 |
|  | rs11191580 | rs11191580 | rs11191580 |
|  | rs11191582 | rs11191582 | rs11191582 |
|  | rs74233809 | rs74233809 | rs74233809 |
|  | rs11191587 | rs11191587 | rs11191587 |
|  | rs12219304 | rs12219304 | rs12219304 |
|  | rs11191593 | rs11191593 | rs11191593 |
|  | rs79237883 | rs79237883 | rs79237883 |
|  | rs34747231 | rs34747231 | rs34747231 |
|  | rs77420391 | rs77420391 | rs77420391 |
|  | rs11191607 | rs11191607 | rs11191607 |
|  | rs11567758 | rs11567758 | rs11567758 |
|  | rs78775782 | rs78775782 | rs78775782 |
|  | rs79059851 | rs79059851 | rs79059851 |
|  | rs77335224 | rs77335224 |  |
|  | rs17878846 | rs17878846 |  |
|  | rs7098825 | rs7098825 |  |
|  | rs11191425 | rs11191425 |  |
|  | rs4409766 | rs4409766 |  |
|  | rs3824754 | rs3824754 |  |
|  | rs11191416 | rs11191416 |  |
|  | rs3824755 | rs3824755 |  |
|  | rs1004467 | rs1004467 |  |
|  | rs17115100 | rs17115100 |  |
|  | rs12416331 | rs12416331 |  |
|  | rs12414028 | rs12414028 |  |
|  | rs11191447 |  |  |
|  |  |  |  |
| ***RAI1-PEMT-RASD1*/rs12936587^a,b^** | rs12936587 | rs12936587 | rs12936587 |
|  | rs12449964 | rs12449964 |  |
|  | rs9630706 |  |  |
|  | rs12945496 |  |  |
|  | rs9913096 |  |  |
|  | rs12602348 |  |  |
|  | rs9900673 |  |  |
|  |  |  |  |
| ***VAMP5-VAMP8-GGCX*/rs1561198** | rs1561198 | rs1561198 | rs1561198 |
|  | rs6738645 | rs6738645 |  |
|  | rs6547621 | rs6547621 |  |
|  | rs7605975 | rs7605975 |  |
|  | rs1078004 | rs1078004 |  |
|  | rs2028900 | rs2028900 |  |
|  | rs6743030 | rs6743030 |  |
|  | rs10179195 | rs10179195 |  |
|  | rs6705971 | rs6705971 |  |
|  | rs6705839 | rs6705839 |  |
|  | rs2366639 | rs2366639 |  |
|  | rs1562323 | rs1562323 |  |
|  | rs1010 | rs1010 |  |
|  | rs1058588 | rs1058588 |  |
|  | rs1009 | rs1009 |  |
|  | rs1972297 | rs1972297 |  |
|  | rs6757263 | rs6757263 |  |
|  | rs1446668 | rs1446668 |  |
|  | rs3755015 | rs3755015 |  |
|  | rs3755014 | rs3755014 |  |
|  | rs10175792 | rs10175792 |  |
|  | rs1348818 |  |  |
|  | rs3731827 |  |  |
|  | rs3770098 |  |  |
|  | rs6547624 |  |  |
|  | rs10198569 |  |  |
|  | rs10187424 |  |  |
|  | rs10172544 |  |  |
|  | rs12473819 |  |  |
|  | rs59877521 |  |  |
|  | rs17026396 |  |  |
|  | rs6714709 |  |  |
|  | rs6547620 |  |  |
|  | rs6739015 |  |  |
|  | rs2166529 |  |  |
|  | rs7591175 |  |  |
|  | rs6733913 |  |  |
|  | rs6719046 |  |  |
|  | rs2044474 |  |  |
|  | rs35215812 |  |  |
|  | rs35565292 |  |  |
|  | rs12714145 |  |  |
|  | rs2886722 |  |  |
|  |  |  |  |
| ***PPAP2B*/rs17114036^a,b^** | rs17114036 | rs17114036 | rs17114036 |
|  | rs72664318 | rs72664318 | rs72664318 |
|  | rs55694910 | rs55694910 |  |
|  | rs9970807 | rs9970807 |  |
|  | rs17114046 | rs17114046 |  |
|  | rs56186267 | rs56186267 |  |
|  | rs72664341 | rs72664341 |  |
|  | rs56322312 | rs56322312 |  |
|  | rs72664304 |  |  |
|  | rs72664303 |  |  |
|  | rs6588635 |  |  |
|  | rs1815487 |  |  |
|  | rs6588634 |  |  |
|  | rs6421496 |  |  |
|  | rs55869368 |  |  |
|  | rs72664335 |  |  |
|  | rs56348932 |  |  |
|  | rs72664354 |  |  |
|  | rs2404715 |  |  |
|  | rs56170783 |  |  |
|  | rs72664324 |  |  |
|  | rs4634932 |  |  |
|  |  |  |  |
| ***MIA3*/rs17465637** | rs17465637 | rs17465637 | rs17465637 |
|  | rs17011681 | rs17011681 | rs17011681 |
|  | rs17163358 | rs17163358 | rs17163358 |
|  | rs17163363 | rs17163363 | rs17163363 |
|  | rs2133189 | rs2133189 | rs2133189 |
|  | rs17465940 | rs17465940 | rs17465940 |
|  | rs67180937 | rs67180937 |  |
|  | rs17163360 | rs17163360 |  |
|  | rs2291834 | rs2291834 |  |
|  | rs35700460 | rs35700460 |  |
|  | rs10495198 | rs10495198 |  |
|  | rs1133220 | rs1133220 |  |
|  | rs17465982 | rs17465982 |  |
|  | rs2291832 | rs2291832 |  |
|  | rs2133187 | rs2133187 |  |
|  | rs17163345 | rs17163345 |  |
|  | rs17163313 | rs17163313 |  |
|  | rs17163301 | rs17163301 |  |
|  | rs2378584 | rs2378584 |  |
|  | rs4575092 |  |  |
|  | rs4846384 |  |  |
|  | rs4846770 |  |  |
|  | rs17532708 |  |  |
|  | rs12043288 |  |  |
|  | rs3748626 |  |  |
|  | rs3002126 |  |  |
|  | rs35626308 |  |  |
|  |  |  |  |
| ***FURIN-FES*/rs17514846** | rs17514846 | rs17514846 | rs17514846 |
|  | rs8039305 | rs8039305 |  |
|  | rs6224 | rs6224 |  |
|  | rs2071382 | rs2071382 |  |
|  | rs11539637 | rs11539637 |  |
|  | rs7177338 | rs7177338 |  |
|  | rs1894401 | rs1894401 |  |
|  | rs7183988 |  |  |
|  |  |  |  |
| ***ANKS1A*/rs17609940** | rs17609940 | rs17609940 | rs17609940 |
|  | rs12154140 | rs12154140 | rs12154140 |
|  | rs12154189 | rs12154189 | rs12154189 |
|  | rs2077750 | rs2077750 | rs2077750 |
|  | rs12215331 | rs12215331 |  |
|  | rs820082 | rs820082 |  |
|  | rs12197124 | rs12197124 |  |
|  | rs4276486 | rs4276486 |  |
|  | rs12206298 | rs12206298 |  |
|  | rs12204265 | rs12204265 |  |
|  | rs12202483 | rs12202483 |  |
|  | rs62402708 | rs62402708 |  |
|  | rs10456428 |  |  |
|  | rs7742443 |  |  |
|  | rs10947525 |  |  |
|  | rs12205331 |  |  |
|  | rs2104332 |  |  |
|  | rs12203818 |  |  |
|  | rs2091074 |  |  |
|  | rs12193946 |  |  |
|  |  |  |  |
| ***HDAC9*/rs2023938** | rs2023938 | rs2023938 | rs2023938 |
|  | rs2023937 | rs2023937 | rs2023937 |
|  | rs11984041 | rs11984041 | rs11984041 |
|  | rs7792656 |  |  |
|  | rs10245779 |  |  |
|  | rs10255384 |  |  |
|  | rs7783974 |  |  |
|  | rs7784712 |  |  |
|  | rs2526619 |  |  |
|  |  |  |  |
| ***CXCL12/*rs2047009** | rs2047009 | rs2047009 | rs2047009 |
|  | rs7903121 | rs7903121 | rs7903121 |
|  | rs11238818 | rs11238818 | rs11238818 |
|  | rs11238817 | rs11238817 | rs11238817 |
|  | rs10899973 | rs10899973 | rs10899973 |
|  | rs9332446 | rs9332446 | rs9332446 |
|  | rs955584 | rs955584 |  |
|  | rs10899971 | rs10899971 |  |
|  | rs1873757 | rs1873757 |  |
|  | rs11238822 | rs11238822 |  |
|  | rs12570314 | rs12570314 |  |
|  | rs10899970 | rs10899970 |  |
|  | rs1352999 | rs1352999 |  |
|  | rs4948815 | rs4948815 |  |
|  | rs10793516 | rs10793516 |  |
|  | rs10899965 | rs10899965 |  |
|  | rs7478408 | rs7478408 |  |
|  | rs1873756 | rs1873756 |  |
|  | rs10793515 | rs10793515 |  |
|  | rs7091447 | rs7091447 |  |
|  | rs4948591 | rs4948591 |  |
|  | rs4948590 | rs4948590 |  |
|  | rs10793511 | rs10793511 |  |
|  | rs1472168 | rs1472168 |  |
|  | rs11238808 | rs11238808 |  |
|  | rs10899963 |  |  |
|  | rs7090343 |  |  |
|  | rs2818904 |  |  |
|  | rs2624695 |  |  |
|  | rs2128363 |  |  |
|  | rs2624694 |  |  |
|  | rs2639463 |  |  |
|  | rs10793517 |  |  |
|  | rs4948818 |  |  |
|  | rs2804029 |  |  |
|  | rs2804027 |  |  |
|  |  |  |  |
| ***SLC22A3-LPAL2-LPA*/rs2048327** | rs2048327 | rs2048327 | rs2048327 |
|  | rs9355288 | rs9355288 | rs9355288 |
|  | rs2292334 | rs2292334 | rs2292334 |
|  | rs7769879 | rs7769879 |  |
|  | rs3918285 | rs3918285 |  |
|  | rs3918286 | rs3918286 |  |
|  | rs1810126 | rs1810126 |  |
|  | rs3088442 | rs3088442 |  |
|  | rs388170 | rs388170 |  |
|  | rs3106164 | rs3106164 |  |
|  | rs2063347 | rs2063347 |  |
|  | rs3106162 | rs3106162 |  |
|  | rs2063346 | rs2063346 |  |
|  | rs2661839 | rs2661839 |  |
|  | rs9365164 | rs9365164 |  |
|  | rs3123636 |  |  |
|  | rs7758229 |  |  |
|  | rs10455782 |  |  |
|  |  |  |  |
| ***ApoE-ApoC1*/rs2075650** | rs2075650 | rs2075650 | rs2075650 |
|  | rs34404554 | rs34404554 | rs34404554 |
|  | rs11556505 | rs11556505 | rs11556505 |
|  | rs71352238 | rs71352238 |  |
|  | rs12972156 |  |  |
|  | rs34342646 |  |  |
|  | rs12972970 |  |  |
|  |  |  |  |
| ***LIPA*/rs2246833** | rs2246833 | rs2246833 | rs2246833 |
|  | rs2246942 | rs2246942 | rs2246942 |
|  | rs1412444 | rs1412444 | rs1412444 |
|  | rs2250644 | rs2250644 | rs2250644 |
|  | rs1412445 | rs1412445 | rs1412445 |
|  | rs2246941 | rs2246941 |  |
|  | rs1332329 | rs1332329 |  |
|  | rs1332328 | rs1332328 |  |
|  | rs2246828 |  |  |
|  | rs1051338 |  |  |
|  | rs2250645 |  |  |
|  | rs2243547 |  |  |
|  | rs1332327 |  |  |
|  |  |  |  |
| ***ZEB2-AC074093.1*/rs2252641** | rs1976974 | rs1976974 | rs1976974 |
|  | rs1830319 | rs1830319 | rs1830319 |
|  | rs13028626 | rs13028626 | rs13028626 |
|  | rs1852687 | rs1852687 |  |
|  | rs7592365 | rs7592365 |  |
|  | rs10928240 | rs10928240 |  |
|  | rs7593336 | rs7593336 |  |
|  | rs2890769 | rs2890769 |  |
|  | rs2381687 | rs2381687 |  |
|  | rs1106909 | rs1106909 |  |
|  | rs13408842 | rs13408842 |  |
|  | rs12618916 | rs12618916 |  |
|  | rs28709970 | rs28709970 |  |
|  | rs787428 |  |  |
|  | rs787429 |  |  |
|  | rs1852685 |  |  |
|  | rs2381686 |  |  |
|  | rs2252641 |  |  |
|  | rs10192407 |  |  |
|  | rs4662414 |  |  |
|  | rs1852683 |  |  |
|  | rs1830321 |  |  |
|  |  |  |  |
| ***SMG6*/rs2281727** | rs2281727 | rs2281727 | rs2281727 |
|  | rs11655813 | rs11655813 | rs11655813 |
|  | rs9895551 | rs9895551 | rs9895551 |
|  | rs62069332 | rs62069332 | rs62069332 |
|  | rs9908888 | rs9908888 | rs9908888 |
|  | rs9900379 | rs9900379 | rs9900379 |
|  | rs6503324 | rs6503324 | rs6503324 |
|  | rs8076939 | rs8076939 | rs8076939 |
|  | rs9897160 | rs9897160 |  |
|  | rs12602764 | rs12602764 |  |
|  | rs12603057 | rs12603057 |  |
|  | rs12601834 | rs12601834 |  |
|  | rs1231209 | rs1231209 |  |
|  | rs1231206 | rs1231206 |  |
|  | rs9893573 | rs9893573 |  |
|  | rs216172 | rs216172 |  |
|  | rs143499 | rs143499 |  |
|  | rs9896535 | rs9896535 |  |
|  | rs216212 | rs216212 |  |
|  | rs7217226 | rs7217226 |  |
|  | rs11078883 | rs11078883 |  |
|  | rs10852932 | rs10852932 |  |
|  | rs216219 | rs216219 |  |
|  | rs216220 | rs216220 |  |
|  | rs216223 | rs216223 |  |
|  | rs9899193 | rs9899193 |  |
|  | rs12943566 | rs12943566 |  |
|  | rs7406247 | rs7406247 |  |
|  | rs12453323 | rs12453323 |  |
|  | rs12938295 | rs12938295 |  |
|  | rs422632 | rs422632 |  |
|  | rs7213232 | rs7213232 |  |
|  | rs216176 | rs216176 |  |
|  | rs216179 | rs216179 |  |
|  | rs1122645 | rs1122645 |  |
|  | rs216180 | rs216180 |  |
|  | rs4790071 | rs4790071 |  |
|  | rs4790072 | rs4790072 |  |
|  | rs4790321 | rs4790321 |  |
|  | rs216183 | rs216183 |  |
|  | rs11657644 | rs11657644 |  |
|  | rs11872068 | rs11872068 |  |
|  | rs11869805 | rs11869805 |  |
|  | rs6502155 | rs6502155 |  |
|  | rs9905529 | rs9905529 |  |
|  | rs8065650 | rs8065650 |  |
|  | rs8066372 | rs8066372 |  |
|  | rs8077545 | rs8077545 |  |
|  | rs749240 | rs749240 |  |
|  | rs216189 | rs216189 |  |
|  | rs216190 | rs216190 |  |
|  | rs8074850 | rs8074850 |  |
|  | rs441750 | rs441750 |  |
|  | rs403553 | rs403553 |  |
|  | rs4790325 | rs4790325 |  |
|  | rs452363 | rs452363 |  |
|  | rs216209 | rs216209 |  |
|  | rs216206 | rs216206 |  |
|  | rs216204 | rs216204 |  |
|  | rs170045 | rs170045 |  |
|  | rs12949991 | rs12949991 |  |
|  | rs216202 | rs216202 |  |
|  | rs170044 | rs170044 |  |
|  | rs177567 | rs177567 |  |
|  | rs216201 | rs216201 |  |
|  | rs394752 | rs394752 |  |
|  | rs375245 | rs375245 |  |
|  | rs216198 | rs216198 |  |
|  | rs9908972 | rs9908972 |  |
|  | rs7212249 | rs7212249 |  |
|  | rs12950555 | rs12950555 |  |
|  | rs9909895 | rs9909895 |  |
|  | rs11078018 | rs11078018 |  |
|  | rs11078019 | rs11078019 |  |
|  | rs4790887 | rs4790887 |  |
|  | rs216217 | rs216217 |  |
|  | rs216218 | rs216218 |  |
|  | rs216222 | rs216222 |  |
|  | rs216178 | rs216178 |  |
|  | rs216191 | rs216191 |  |
|  | rs11078024 | rs11078024 |  |
|  | rs432200 | rs432200 |  |
|  | rs12941621 | rs12941621 |  |
|  | rs12941836 | rs12941836 |  |
|  | rs11078865 |  |  |
|  | rs11651451 |  |  |
|  | rs7217687 |  |  |
|  | rs2169357 |  |  |
|  | rs9897624 |  |  |
|  | rs59428454 |  |  |
|  | rs28524880 |  |  |
|  | rs216213 |  |  |
|  | rs9891227 |  |  |
|  | rs404392 |  |  |
|  | rs4523957 |  |  |
|  | rs1002135 |  |  |
|  | rs57307236 |  |  |
|  | rs216200 |  |  |
|  | rs216193 |  |  |
|  | rs2209073 |  |  |
|  | rs2224770 |  |  |
|  | rs12603592 |  |  |
|  | rs12450354 |  |  |
|  | rs1532292 |  |  |
|  | rs6503321 |  |  |
|  | rs9906500 |  |  |
|  | rs1563966 |  |  |
|  |  |  |  |
| ***KIAA1462*/rs2505083** | rs2505083 | rs2505083 | rs2505083 |
|  | rs765906 | rs765906 |  |
|  | rs2487928 | rs2487928 |  |
|  | rs1887318 |  |  |
|  | rs2478835 |  |  |
|  | rs3739998 |  |  |
|  | rs2487927 |  |  |
|  | rs2478839 |  |  |
|  | rs2505084 |  |  |
|  |  |  |  |
| ***LPL*/rs264** | rs264 | rs264 | rs264 |
|  | rs256 | rs256 | rs256 |
|  | rs271 | rs271 |  |
|  | rs255 | rs255 |  |
|  | rs254 | rs254 |  |
|  | rs3779788 |  |  |
|  |  |  |  |
| ***SLC22A4-SLC22A5*/rs273909** | rs273909 | rs273909 | rs273909 |
|  | rs17689550 | rs17689550 | rs17689550 |
|  |  |  |  |
| ***HHIPL1*/rs2895811** | rs2895811 | rs2895811 | rs2895811 |
|  | rs12436072 | rs12436072 | rs12436072 |
|  | rs34668196 | rs34668196 | rs34668196 |
|  | rs10145905 | rs10145905 | rs10145905 |
|  | rs2400762 | rs2400762 | rs2400762 |
|  | rs28391527 | rs28391527 | rs28391527 |
|  | rs4905878 | rs4905878 | rs4905878 |
|  | rs10139462 | rs10139462 | rs10139462 |
|  | rs7146731 | rs7146731 |  |
|  | rs10149871 | rs10149871 |  |
|  | rs17562391 | rs17562391 |  |
|  | rs12432679 | rs12432679 |  |
|  | rs4624107 | rs4624107 |  |
|  | rs12431602 | rs12431602 |  |
|  | rs4990377 | rs4990377 |  |
|  | rs7145262 | rs7145262 |  |
|  | rs7158073 | rs7158073 |  |
|  |  |  |  |
| ***TRIB1*/rs2954029^a,b^** | rs2954029 | rs2954029 | rs2954029 |
|  | rs2954031 | rs2954031 | rs2954031 |
|  | rs10808546 | rs10808546 | rs10808546 |
|  | rs2980860 | rs2980860 | rs2980860 |
|  | rs2954027 | rs2954027 | rs2954027 |
|  | rs2980875 | rs2980875 | rs2980875 |
|  | rs2980871 | rs2980871 |  |
|  | rs2980869 | rs2980869 |  |
|  | rs2980868 | rs2980868 |  |
|  | rs17321515 | rs17321515 |  |
|  | rs2980882 | rs2980882 |  |
|  | rs6982636 | rs6982636 |  |
|  | rs2001844 | rs2001844 |  |
|  | rs2980853 | rs2980853 |  |
|  | rs2954019 | rs2954019 |  |
|  | rs2980855 | rs2980855 |  |
|  | rs2980856 | rs2980856 |  |
|  | rs2954022 | rs2954022 |  |
|  | rs2980854 | rs2980854 |  |
|  |  |  |  |
| ***SH2B3*/rs3184504** | rs3184504 | rs3184504 | rs3184504 |
|  | rs4766578 | rs4766578 |  |
|  | rs10774625 | rs10774625 |  |
|  | rs7137828 | rs7137828 |  |
|  | rs653178 |  |  |
|  |  |  |  |
| ***CDKN2BAS1*/rs3217992** | rs3217992 | rs3217992 | rs3217992 |
|  | rs2069416 | rs2069416 |  |
|  | rs10811641 |  |  |
|  | rs10738604 |  |  |
|  | rs7028268 |  |  |
|  |  |  |  |
| ***PLG*/rs4252120^b,c^** | rs4252120 | rs4252120 | rs4252120 |
|  | rs4252117 | rs4252117 | rs4252117 |
|  | rs4252114 | rs4252114 | rs4252114 |
|  | rs9458016 | rs9458016 | rs9458016 |
|  | rs1321197 | rs1321197 | rs1321197 |
|  | rs9456578 | rs9456578 | rs9456578 |
|  | rs4252125 | rs4252125 | rs4252125 |
|  | rs4252126 | rs4252126 | rs4252126 |
|  | rs4252130 | rs4252130 | rs4252130 |
|  | rs4252134 | rs4252134 | rs4252134 |
|  | rs9458019 | rs9458019 | rs9458019 |
|  | rs4252135 | rs4252135 | rs4252135 |
|  | rs9458020 | rs9458020 | rs9458020 |
|  | rs56093624 | rs56093624 | rs56093624 |
|  | rs4252150 | rs4252150 | rs4252150 |
|  | rs4252151 | rs4252151 | rs4252151 |
|  | rs4252165 | rs4252165 | rs4252165 |
|  | rs1897108 | rs1897108 | rs1897108 |
|  | rs13231 | rs13231 | rs13231 |
|  | rs4252109 | rs4252109 | rs4252109 |
|  | rs9458017 | rs9458017 | rs9458017 |
|  | rs4252096 | rs4252096 | rs4252096 |
|  | rs4252093 | rs4252093 | rs4252093 |
|  | rs4252090 | rs4252090 | rs4252090 |
|  | rs4252087 | rs4252087 | rs4252087 |
|  | rs1853018 | rs1853018 | rs1853018 |
|  | rs1972748 | rs1972748 | rs1972748 |
|  | rs4252072 | rs4252072 | rs4252072 |
|  | rs28402939 | rs28402939 | rs28402939 |
|  | rs4252086 | rs4252086 | rs4252086 |
|  | rs4252107 |  |  |
|  | rs4252076 |  |  |
|  | rs4252181 |  |  |
|  | rs56262039 |  |  |
|  | rs9456580 |  |  |
|  | rs4252082 |  |  |
|  | rs62439805 |  |  |
|  | rs9458022 |  |  |
|  | rs4252066 |  |  |
|  | rs9458012 |  |  |
|  | rs62436702 |  |  |
|  |  |  |  |
| ***ApoE-ApoC1*/rs445925** | rs445925 | rs445925 | rs445925 |
|  | rs72654473 | rs72654473 | rs72654473 |
|  |  |  |  |
| ***UBE2Z*/rs46522^b,c^** | rs46522 | rs46522 | rs46522 |
|  | rs58838744 | rs58838744 |  |
|  | rs8182364 | rs8182364 |  |
|  | rs318091 | rs318091 |  |
|  | rs61576918 | rs61576918 |  |
|  | rs318090 | rs318090 |  |
|  | rs4294857 | rs4294857 |  |
|  | rs62075820 | rs62075820 |  |
|  | rs9912829 | rs9912829 |  |
|  | rs12453374 | rs12453374 |  |
|  | rs62075818 | rs62075818 |  |
|  | rs12453394 | rs12453394 |  |
|  | rs62075838 | rs62075838 |  |
|  | rs62075839 | rs62075839 |  |
|  | rs4255820 | rs4255820 |  |
|  | rs903567 | rs903567 |  |
|  | rs28409394 | rs28409394 |  |
|  | rs962272 | rs962272 |  |
|  | rs55771415 | rs55771415 |  |
|  | rs2112617 | rs2112617 |  |
|  | rs2088139 | rs2088139 |  |
|  | rs4793991 | rs4793991 |  |
|  | rs318096 | rs318096 |  |
|  | rs318095 | rs318095 |  |
|  | rs832410 | rs832410 |  |
|  | rs15563 | rs15563 |  |
|  | rs17635252 | rs17635252 |  |
|  | rs9904645 | rs9904645 |  |
|  | rs4793992 | rs4793992 |  |
|  | rs56046215 | rs56046215 |  |
|  | rs1008834 | rs1008834 |  |
|  | rs4399576 | rs4399576 |  |
|  | rs17708633 | rs17708633 |  |
|  | rs1994970 | rs1994970 |  |
|  | rs9747646 | rs9747646 |  |
|  | rs62075852 | rs62075852 |  |
|  | rs12601858 | rs12601858 |  |
|  | rs4793995 | rs4793995 |  |
|  | rs4793996 | rs4793996 |  |
|  | rs4793998 | rs4793998 |  |
|  | rs4794000 | rs4794000 |  |
|  | rs58591767 | rs58591767 |  |
|  | rs59270107 | rs59270107 |  |
|  | rs62078370 | rs62078370 |  |
|  | rs118135644 | rs118135644 |  |
|  | rs11079844 | rs11079844 |  |
|  | rs62078372 | rs62078372 |  |
|  | rs12602933 | rs12602933 |  |
|  | rs4794003 | rs4794003 |  |
|  | rs12602746 | rs12602746 |  |
|  | rs12603969 | rs12603969 |  |
|  | rs12601955 | rs12601955 |  |
|  | rs2291725 | rs2291725 |  |
|  | rs28517720 | rs28517720 |  |
|  | rs62075844 | rs62075844 |  |
|  | rs2643361 | rs2643361 |  |
|  | rs4793605 | rs4793605 |  |
|  | rs2291726 | rs2291726 |  |
|  | rs62078385 | rs62078385 |  |
|  | rs937301 | rs937301 |  |
|  | rs3848460 | rs3848460 |  |
|  | rs12601072 | rs12601072 |  |
|  | rs60708039 | rs60708039 |  |
|  | rs1057897 | rs1057897 |  |
|  | rs519537 | rs519537 |  |
|  | rs12601672 | rs12601672 |  |
|  | rs80032154 | rs80032154 |  |
|  | rs46521 | rs46521 |  |
|  | rs318093 | rs318093 |  |
|  | rs2546491 | rs2546491 |  |
|  | rs957557 | rs957557 |  |
|  | rs1057902 | rs1057902 |  |
|  | rs12602179 | rs12602179 |  |
|  | rs2270574 | rs2270574 |  |
|  | rs62075824 |  |  |
|  | rs3895874 |  |  |
|  | rs4794004 |  |  |
|  | rs55724082 |  |  |
|  | rs4793997 |  |  |
|  | rs170319 |  |  |
|  | rs9894239 |  |  |
|  | rs3744608 |  |  |
|  | rs4378658 |  |  |
|  | rs1985785 |  |  |
|  | rs12950328 |  |  |
|  | rs595767 |  |  |
|  | rs62078384 |  |  |
|  |  |  |  |
| ***COL4A1-COL4A2*/rs4773144** | rs4773144 | rs4773144 | rs4773144 |
|  | rs4773143 | rs4773143 |  |
|  | rs7986871 | rs7986871 |  |
|  | rs3809346 | rs3809346 |  |
|  |  |  |  |
| ***IL6R*/rs4845625** | rs4845625 | rs4845625 | rs4845625 |
|  | rs6689393 | rs6689393 |  |
|  | rs4553185 | rs4553185 |  |
|  | rs6667434 | rs6667434 |  |
|  | rs59632925 | rs59632925 |  |
|  | rs4845619 | rs4845619 |  |
|  | rs7553796 | rs7553796 |  |
|  | rs7549338 | rs7549338 |  |
|  | rs7549250 | rs7549250 |  |
|  | rs4845371 |  |  |
|  | rs11265612 |  |  |
|  | rs6694817 |  |  |
|  | rs6687726 |  |  |
|  | rs4845618 |  |  |
|  | rs6689306 |  |  |
|  |  |  |  |
| ***CXCL12*/rs501120^a,b^** | rs501120 | rs501120 | rs501120 |
|  | rs503859 | rs503859 | rs503859 |
|  | rs504799 | rs504799 | rs504799 |
|  | rs670056 | rs670056 | rs670056 |
|  | rs671765 | rs671765 | rs671765 |
|  | rs579058 | rs579058 | rs579058 |
|  | rs534079 | rs534079 | rs534079 |
|  | rs622956 | rs622956 | rs622956 |
|  | rs915083 | rs915083 | rs915083 |
|  | rs554568 | rs554568 | rs554568 |
|  | rs554565 | rs554565 | rs554565 |
|  | rs559580 | rs559580 | rs559580 |
|  | rs687175 | rs687175 | rs687175 |
|  | rs607760 | rs607760 | rs607760 |
|  | rs522293 | rs522293 | rs522293 |
|  | rs605445 | rs605445 | rs605445 |
|  | rs604674 | rs604674 | rs604674 |
|  | rs518594 | rs518594 | rs518594 |
|  | rs665855 | rs665855 | rs665855 |
|  | rs487465 | rs487465 | rs487465 |
|  | rs573141 | rs573141 | rs573141 |
|  | rs634963 | rs634963 | rs634963 |
|  | rs479596 | rs479596 | rs479596 |
|  | rs620828 | rs620828 | rs620828 |
|  | rs620356 | rs620356 | rs620356 |
|  | rs607363 | rs607363 | rs607363 |
|  | rs694425 | rs694425 | rs694425 |
|  | rs527785 | rs527785 | rs527785 |
|  | rs493874 | rs493874 | rs493874 |
|  | rs552794 | rs552794 | rs552794 |
|  | rs1746047 | rs1746047 | rs1746047 |
|  | rs1746048 | rs1746048 | rs1746048 |
|  | rs605425 | rs605425 |  |
|  | rs2576354 | rs2576354 |  |
|  | rs2576355 | rs2576355 |  |
|  | rs528668 | rs528668 |  |
|  | rs684521 | rs684521 |  |
|  | rs535949 | rs535949 |  |
|  | rs559469 | rs559469 |  |
|  | rs492152 | rs492152 |  |
|  | rs2505732 | rs2505732 |  |
|  | rs494045 | rs494045 |  |
|  | rs1632484 | rs1632484 |  |
|  | rs1746049 | rs1746049 |  |
|  | rs1746050 | rs1746050 |  |
|  | rs1746052 | rs1746052 |  |
|  | rs1657345 | rs1657345 |  |
|  | rs1657344 | rs1657344 |  |
|  | rs607609 |  |  |
|  | rs642222 |  |  |
|  | rs607592 |  |  |
|  | rs510785 |  |  |
|  | rs513391 |  |  |
|  | rs622472 |  |  |
|  | rs473501 |  |  |
|  | rs474281 |  |  |
|  | rs535176 |  |  |
|  | rs541483 |  |  |
|  | rs683297 |  |  |
|  | rs684196 |  |  |
|  | rs684666 |  |  |
|  | rs498810 |  |  |
|  |  |  |  |
| ***APOB*/rs515135^a,b^** | rs515135 | rs515135 | rs515135 |
|  | rs563290 | rs563290 | rs563290 |
|  | rs562338 | rs562338 | rs562338 |
|  | rs581411 | rs581411 | rs581411 |
|  | rs668948 | rs668948 | rs668948 |
|  | rs541041 | rs541041 | rs541041 |
|  | rs548145 | rs548145 |  |
|  | rs580889 |  |  |
|  | rs481069 |  |  |
|  | rs35913552 |  |  |
|  | rs34722314 |  |  |
|  | rs12714264 |  |  |
|  |  |  |  |
| ***ABO*/rs579459** | rs579459 | rs579459 | rs579459 |
|  | rs649129 | rs649129 | rs649129 |
|  | rs651007 | rs651007 | rs651007 |
|  | rs495828 | rs495828 | rs495828 |
|  | rs635634 | rs635634 | rs635634 |
|  | rs600038 | rs600038 |  |
|  | rs532436 | rs532436 |  |
|  | rs115478735 | rs115478735 |  |
|  | rs507666 | rs507666 |  |
|  | rs2519093 | rs2519093 |  |
|  |  |  |  |
| ***SORT1*/rs602633^b,c^** | rs602633 | rs602633 | rs602633 |
|  | rs583104 | rs583104 | rs583104 |
|  | rs1277930 | rs1277930 | rs1277930 |
|  | rs599839 | rs599839 | rs599839 |
|  | rs646776 |  |  |
|  | rs629301 |  |  |
|  | rs12740374 |  |  |
|  | rs7528419 |  |  |
|  | rs660240 |  |  |
|  |  |  |  |
| ***ABCG5-ABCG8*/rs6544713** | rs6544713 | rs6544713 | rs6544713 |
|  | rs4245791 | rs4245791 | rs4245791 |
|  | rs4299376 | rs4299376 | rs4299376 |
|  | rs4952688 |  |  |
|  | rs6544717 |  |  |
|  | rs4603816 |  |  |
|  | rs6755809 |  |  |
|  | rs4953026 |  |  |
|  |  |  |  |
| ***WDR12*/rs6725887** | rs6725887 | rs6725887 | rs6725887 |
|  | rs6722332 | rs6722332 | rs6722332 |
|  | rs72934749 | rs72934749 | rs72934749 |
|  | rs72934745 | rs72934745 | rs72934745 |
|  | rs115953525 | rs115953525 | rs115953525 |
|  | rs72934751 | rs72934751 | rs72934751 |
|  | rs6738618 | rs6738618 | rs6738618 |
|  | rs72934753 | rs72934753 | rs72934753 |
|  | rs72934740 | rs72934740 | rs72934740 |
|  | rs79539678 | rs79539678 | rs79539678 |
|  | rs72934738 | rs72934738 | rs72934738 |
|  | rs72934737 | rs72934737 | rs72934737 |
|  | rs72934735 | rs72934735 | rs72934735 |
|  | rs72934732 | rs72934732 | rs72934732 |
|  | rs116382857 | rs116382857 | rs116382857 |
|  | rs77268589 | rs77268589 | rs77268589 |
|  | rs6435169 | rs6435169 | rs6435169 |
|  | rs7582720 | rs7582720 | rs7582720 |
|  | rs6723704 | rs6723704 | rs6723704 |
|  | rs72934729 | rs72934729 | rs72934729 |
|  | rs4510208 | rs4510208 | rs4510208 |
|  | rs3845800 | rs3845800 | rs3845800 |
|  | rs7560547 | rs7560547 | rs7560547 |
|  | rs72934763 | rs72934763 | rs72934763 |
|  | rs72934764 | rs72934764 | rs72934764 |
|  | rs72934765 | rs72934765 | rs72934765 |
|  | rs77931721 | rs77931721 | rs77931721 |
|  | rs35212307 | rs35212307 | rs35212307 |
|  | rs72934767 | rs72934767 | rs72934767 |
|  | rs7605484 | rs7605484 | rs7605484 |
|  | rs114395475 | rs114395475 | rs114395475 |
|  | rs72936830 | rs72936830 | rs72936830 |
|  | rs115396314 | rs115396314 | rs115396314 |
|  | rs72936834 | rs72936834 | rs72936834 |
|  | rs114899426 | rs114899426 | rs114899426 |
|  | rs72936838 | rs72936838 | rs72936838 |
|  | rs114155121 | rs114155121 | rs114155121 |
|  | rs72936842 | rs72936842 | rs72936842 |
|  | rs72936846 | rs72936846 | rs72936846 |
|  | rs72936847 | rs72936847 | rs72936847 |
|  | rs72936852 | rs72936852 | rs72936852 |
|  | rs72936856 | rs72936856 | rs72936856 |
|  | rs72934715 | rs72934715 | rs72934715 |
|  | rs72934714 | rs72934714 | rs72934714 |
|  | rs72934711 | rs72934711 | rs72934711 |
|  | rs72934710 | rs72934710 | rs72934710 |
|  | rs72934707 | rs72934707 | rs72934707 |
|  | rs72934706 | rs72934706 | rs72934706 |
|  | rs72934704 | rs72934704 | rs72934704 |
|  | rs72936862 | rs72936862 | rs72936862 |
|  | rs114527590 | rs114527590 | rs114527590 |
|  | rs72936869 | rs72936869 | rs72936869 |
|  | rs72936870 | rs72936870 | rs72936870 |
|  | rs72932793 | rs72932793 | rs72932793 |
|  | rs72936872 | rs72936872 | rs72936872 |
|  | rs72932791 | rs72932791 | rs72932791 |
|  | rs72936873 | rs72936873 | rs72936873 |
|  | rs72932789 | rs72932789 | rs72932789 |
|  | rs72932786 | rs72932786 | rs72932786 |
|  | rs72932784 | rs72932784 | rs72932784 |
|  | rs75324925 | rs75324925 | rs75324925 |
|  | rs72936881 | rs72936881 | rs72936881 |
|  | rs72936882 | rs72936882 | rs72936882 |
|  | rs72932781 | rs72932781 | rs72932781 |
|  | rs114520702 | rs114520702 | rs114520702 |
|  | rs72932780 | rs72932780 | rs72932780 |
|  | rs114393235 | rs114393235 | rs114393235 |
|  | rs72926767 | rs72926767 | rs72926767 |
|  | rs72926769 | rs72926769 | rs72926769 |
|  | rs75166090 | rs75166090 | rs75166090 |
|  | rs72926771 | rs72926771 | rs72926771 |
|  | rs72932777 | rs72932777 | rs72932777 |
|  | rs72932776 | rs72932776 | rs72932776 |
|  | rs72926772 | rs72926772 | rs72926772 |
|  | rs77230711 | rs77230711 | rs77230711 |
|  | rs72926779 | rs72926779 | rs72926779 |
|  | rs72932774 | rs72932774 | rs72932774 |
|  | rs75141346 | rs75141346 | rs75141346 |
|  | rs75869289 | rs75869289 | rs75869289 |
|  | rs79633844 | rs79633844 | rs79633844 |
|  | rs72926781 | rs72926781 | rs72926781 |
|  | rs72932772 | rs72932772 | rs72932772 |
|  | rs72932770 | rs72932770 | rs72932770 |
|  | rs72926782 | rs72926782 | rs72926782 |
|  | rs72932767 | rs72932767 | rs72932767 |
|  | rs72932765 | rs72932765 | rs72932765 |
|  | rs72932763 | rs72932763 | rs72932763 |
|  | rs76298043 | rs76298043 | rs76298043 |
|  | rs115400054 | rs115400054 | rs115400054 |
|  | rs72926783 | rs72926783 | rs72926783 |
|  | rs72926786 | rs72926786 | rs72926786 |
|  | rs72926787 | rs72926787 | rs72926787 |
|  | rs74675536 | rs74675536 | rs74675536 |
|  | rs80087860 | rs80087860 | rs80087860 |
|  | rs116678869 | rs116678869 | rs116678869 |
|  | rs72932753 | rs72932753 | rs72932753 |
|  | rs114702158 | rs114702158 | rs114702158 |
|  | rs72926791 | rs72926791 | rs72926791 |
|  | rs114110842 | rs114110842 | rs114110842 |
|  | rs72932752 | rs72932752 | rs72932752 |
|  | rs114079739 | rs114079739 | rs114079739 |
|  | rs72926793 | rs72926793 | rs72926793 |
|  | rs72926794 | rs72926794 | rs72926794 |
|  | rs72926796 | rs72926796 | rs72926796 |
|  | rs78128841 | rs78128841 | rs78128841 |
|  | rs72932746 | rs72932746 | rs72932746 |
|  | rs72932745 | rs72932745 | rs72932745 |
|  | rs6705330 | rs6705330 | rs6705330 |
|  | rs72926802 | rs72926802 | rs72926802 |
|  | rs72932741 | rs72932741 | rs72932741 |
|  | rs79642273 | rs79642273 | rs79642273 |
|  | rs114123510 | rs114123510 | rs114123510 |
|  | rs74421437 | rs74421437 | rs74421437 |
|  | rs72928605 | rs72928605 | rs72928605 |
|  | rs1541853 | rs1541853 | rs1541853 |
|  | rs72928608 | rs72928608 | rs72928608 |
|  | rs72932737 | rs72932737 | rs72932737 |
|  | rs72928609 | rs72928609 | rs72928609 |
|  | rs72928613 | rs72928613 | rs72928613 |
|  | rs72928620 | rs72928620 | rs72928620 |
|  | rs72932731 | rs72932731 | rs72932731 |
|  | rs72932727 | rs72932727 | rs72932727 |
|  | rs72932725 | rs72932725 | rs72932725 |
|  | rs72932553 | rs72932553 | rs72932553 |
|  | rs72932554 | rs72932554 | rs72932554 |
|  | rs72932557 | rs72932557 | rs72932557 |
|  | rs72932558 | rs72932558 | rs72932558 |
|  | rs72932561 | rs72932561 | rs72932561 |
|  | rs76890136 | rs76890136 | rs76890136 |
|  | rs72932566 | rs72932566 | rs72932566 |
|  | rs72932572 | rs72932572 | rs72932572 |
|  | rs72932573 | rs72932573 | rs72932573 |
|  | rs76461893 | rs76461893 | rs76461893 |
|  | rs72932574 | rs72932574 | rs72932574 |
|  | rs4675310 | rs4675310 | rs4675310 |
|  | rs72932588 | rs72932588 | rs72932588 |
|  | rs72932590 | rs72932590 | rs72932590 |
|  | rs115654617 | rs115654617 | rs115654617 |
|  | rs72934505 | rs72934505 | rs72934505 |
|  | rs72934510 | rs72934510 | rs72934510 |
|  | rs72934512 | rs72934512 | rs72934512 |
|  | rs72934513 | rs72934513 | rs72934513 |
|  | rs78907692 | rs78907692 | rs78907692 |
|  | rs72934514 | rs72934514 | rs72934514 |
|  | rs72934518 | rs72934518 | rs72934518 |
|  | rs72934519 | rs72934519 | rs72934519 |
|  | rs115810193 | rs115810193 | rs115810193 |
|  | rs72934535 | rs72934535 | rs72934535 |
|  | rs72934537 | rs72934537 | rs72934537 |
|  | rs72934545 | rs72934545 | rs72934545 |
|  | rs72934554 | rs72934554 | rs72934554 |
|  | rs72934556 | rs72934556 | rs72934556 |
|  | rs72934563 | rs72934563 | rs72934563 |
|  | rs72934573 | rs72934573 | rs72934573 |
|  | rs72936304 | rs72936304 | rs72936304 |
|  | rs72936309 | rs72936309 | rs72936309 |
|  | rs72936323 | rs72936323 | rs72936323 |
|  | rs72936326 | rs72936326 | rs72936326 |
|  | rs72936332 | rs72936332 | rs72936332 |
|  | rs72936348 | rs72936348 | rs72936348 |
|  | rs72936353 | rs72936353 | rs72936353 |
|  | rs11458630 | rs11458630 | rs11458630 |
|  | rs11678748 | rs11678748 |  |
|  | rs72934760 | rs72934760 |  |
|  | rs72936860 | rs72936860 |  |
|  | rs72936875 | rs72936875 |  |
|  | rs116773016 | rs116773016 |  |
|  | rs114604411 | rs114604411 |  |
|  | rs72932759 | rs72932759 |  |
|  | rs72932723 | rs72932723 |  |
|  | rs72932722 | rs72932722 |  |
|  | rs72932720 | rs72932720 |  |
|  | rs72932716 | rs72932716 |  |
|  | rs72932711 | rs72932711 |  |
|  | rs72932709 | rs72932709 |  |
|  | rs72932707 | rs72932707 |  |
|  | rs2351524 | rs2351524 |  |
|  | rs72934583 | rs72934583 |  |
|  | rs72934601 | rs72934601 |  |
|  | rs72934734 | rs72934734 |  |
|  | rs72934762 | rs72934762 |  |
|  | rs115827549 | rs115827549 |  |
|  | rs115130739 | rs115130739 |  |
|  | rs72936839 | rs72936839 |  |
|  | rs114372659 | rs114372659 |  |
|  | rs72936866 | rs72936866 |  |
|  | rs72936879 | rs72936879 |  |
|  | rs115628302 | rs115628302 |  |
|  | rs76122535 | rs76122535 |  |
|  | rs72926798 | rs72926798 |  |
|  | rs72926799 | rs72926799 |  |
|  | rs72926800 | rs72926800 |  |
|  | rs72928610 | rs72928610 |  |
|  | rs72932556 | rs72932556 |  |
|  | rs72932559 | rs72932559 |  |
|  | rs72932560 | rs72932560 |  |
|  | rs72932575 | rs72932575 |  |
|  | rs72932583 | rs72932583 |  |
|  | rs72934546 | rs72934546 |  |
|  | rs72934550 | rs72934550 |  |
|  | rs72934551 | rs72934551 |  |
|  | rs1141093 |  |  |
|  | rs72932755 |  |  |
|  | rs72934589 |  |  |
|  | rs72934591 |  |  |
|  | rs115600411 |  |  |
|  | rs72938315 |  |  |
|  | rs114139737 |  |  |
|  | rs115194657 |  |  |
|  | rs116426890 |  |  |
|  | rs72926770 |  |  |
|  | rs10932008 |  |  |
|  | rs11586673 |  |  |
|  | rs11636588 |  |  |
|  |  |  |  |
| ***ADAMTS7*/rs7173743^b,c^** | rs7173743 | rs7173743 | rs7173743 |
|  | rs4344704 | rs4344704 | rs4344704 |
|  | rs4567668 | rs4567668 | rs4567668 |
|  | rs11632020 | rs11632020 | rs11632020 |
|  | rs4438276 | rs4438276 | rs4438276 |
|  | rs4420501 | rs4420501 | rs4420501 |
|  | rs28580532 | rs28580532 |  |
|  | rs28694044 | rs28694044 |  |
|  | rs5029904 | rs5029904 |  |
|  | rs11857877 | rs11857877 |  |
|  | rs12232282 | rs12232282 |  |
|  | rs12903542 | rs12903542 |  |
|  | rs7168915 | rs7168915 |  |
|  | rs11632720 |  |  |
|  | rs11639335 |  |  |
|  |  |  |  |
| ***GUCY1A3*/rs7692387** | rs7692387 | rs7692387 | rs7692387 |
|  | rs3796587 | rs3796587 | rs3796587 |
|  | rs2306556 | rs2306556 | rs2306556 |
|  | rs72689147 | rs72689147 | rs72689147 |
|  | rs3796581 | rs3796581 | rs3796581 |
|  | rs11724647 | rs11724647 | rs11724647 |
|  | rs10029150 | rs10029150 |  |
|  | rs56256623 | rs56256623 |  |
|  | rs56329057 | rs56329057 |  |
|  | rs11721947 | rs11721947 |  |
|  | rs6536087 |  |  |
|  | rs6849302 |  |  |
|  | rs115917428 |  |  |
|  | rs3796592 |  |  |
|  | rs12502903 |  |  |
|  | rs10517620 |  |  |
|  |  |  |  |
| ***FLT1*/rs9319428** | rs9319428 | rs9319428 | rs9319428 |
|  | rs9319429 | rs9319429 | rs9319429 |
|  | rs9513097 | rs9513097 | rs9513097 |
|  | rs9508023 | rs9508023 | rs9508023 |
|  | rs7983774 | rs7983774 | rs7983774 |
|  | rs9508025 | rs9508025 | rs9508025 |
|  | rs9508026 | rs9508026 | rs9508026 |
|  | rs17086617 | rs17086617 | rs17086617 |
|  | rs8002951 | rs8002951 | rs8002951 |
|  | rs9513105 | rs9513105 | rs9513105 |
|  | rs9508029 | rs9508029 | rs9508029 |
|  | rs9513107 | rs9513107 | rs9513107 |
|  | rs1555641 | rs1555641 |  |
|  | rs9513095 | rs9513095 |  |
|  | rs2296284 | rs2296284 |  |
|  | rs9513106 | rs9513106 |  |
|  | rs7992068 | rs7992068 |  |
|  |  |  |  |
|  |  |  |  |
| ***PHACTR1*/rs9369640** | rs9369640 | rs9369640 | rs9369640 |
|  | rs6911226 | rs6911226 | rs6911226 |
|  | rs6915983 | rs6915983 | rs6915983 |
|  | rs9395214 | rs9395214 | rs9395214 |
|  | rs2876302 | rs2876302 | rs2876302 |
|  | rs9296512 | rs9296512 | rs9296512 |
|  | rs7760016 | rs7760016 | rs7760016 |
|  | rs7454157 | rs7454157 | rs7454157 |
|  | rs6925904 | rs6925904 | rs6925904 |
|  | rs7750679 | rs7750679 | rs7750679 |
|  | rs12530250 | rs12530250 | rs12530250 |
|  | rs9381494 | rs9381494 | rs9381494 |
|  | rs1332844 | rs1332844 | rs1332844 |
|  | rs2327620 | rs2327620 |  |
|  | rs7751826 | rs7751826 |  |
|  | rs7760527 | rs7760527 |  |
|  | rs2876301 | rs2876301 |  |
|  | rs62389955 | rs62389955 |  |
|  | rs4714955 | rs4714955 |  |
|  | rs9381500 | rs9381500 |  |
|  | rs6458545 | rs6458545 |  |
|  | rs2876303 | rs2876303 |  |
|  | rs9369650 | rs9369650 |  |
|  | rs1953088 | rs1953088 |  |
|  | rs12526453 | rs12526453 |  |
|  | rs7739181 | rs7739181 |  |
|  | rs4711863 |  |  |
|  | rs8180558 |  |  |
|  | rs2327621 |  |  |
|  | rs1014342 |  |  |
|  | rs4714990 |  |  |
|  | rs13197912 |  |  |
|  | rs62386818 |  |  |
|  | rs34343839 |  |  |
|  |  |  |  |
|  |  |  |  |
| ***ZNF259-APOA5-APOA1*/rs964184** | rs964184 | rs964184 | rs964184 |
|  |  |  |  |
| ***PDGFD*/rs974819** | rs974819 | rs974819 | rs974819 |
|  | rs2019090 | rs2019090 |  |
|  | rs11226029 | rs11226029 |  |
|  | rs2128739 |  |  |
|  | rs2839812 |  |  |
|  | rs1384705 |  |  |
|  |  |  |  |
| ***MRAS*/rs9818870** | rs9818870 | rs9818870 | rs9818870 |
|  | rs3732837 | rs3732837 | rs3732837 |
|  | rs2291127 | rs2291127 | rs2291127 |
|  | rs9851766 | rs9851766 | rs9851766 |
|  | rs2279241 | rs2279241 | rs2279241 |
|  | rs2293251 | rs2293251 | rs2293251 |
|  | rs2306374 | rs2306374 | rs2306374 |
|  | rs9872754 | rs9872754 | rs9872754 |
|  | rs13083299 | rs13083299 | rs13083299 |
|  | rs13096934 | rs13096934 | rs13096934 |
|  | rs9864898 | rs9864898 | rs9864898 |
|  | rs12695685 | rs12695685 | rs12695685 |
|  | rs2347252 | rs2347252 | rs2347252 |
|  | rs1199338 | rs1199338 | rs1199338 |
|  | rs13324341 | rs13324341 |  |
|  | rs1199337 | rs1199337 |  |
|  | rs9848655 |  |  |
|  | rs6807945 |  |  |
|  |  |  |  |
| ***KCNE2*/rs9982601** | rs9982601 | rs9982601 | rs9982601 |
|  | rs9980618 | rs9980618 | rs9980618 |
|  | rs9977419 | rs9977419 |  |
|  | rs9976596 |  |  |
|  | rs60687229 |  |  |
|  | rs9305545 |  |  |
|  | rs28451064 |  |  |
|  | rs8131284 |  |  |
|  | rs9978407 |  |  |
|  | rs7278204 |  |  |
|  | rs8131303 |  |  |
|  | rs28593428 |  |  |
|  | rs973754 |  |  |
|  | rs7280612 |  |  |
|  | rs7278845 |  |  |
|  | rs9983490 |  |  |
|  | rs28591415 |  |  |
|  | rs8132042 |  |  |
|  |  |  |  |
|  |  |  |  |
| ***SLC22A3-LPAL2-LPA/***  **rs3798220^a,b,c,d,e^** |  |  |  |
|  |  |  |  |
| ***CDK2BAS1/*rs1333049** | rs1333049 | rs1333049 | rs1333049 |
|  | rs1004638 | rs10116277 | rs10757278 |
|  | rs10116277 | rs10733376 | rs7859362 |
|  | rs10511701 | rs10738607 |  |
|  | rs10733376 | rs10738610 |  |
|  | rs10738607 | rs10757272 |  |
|  | rs10738609 | rs10757274 |  |
|  | rs10738610 | rs10757278 |  |
|  | rs10757269 | rs1333042 |  |
|  | rs10757272 | rs1333043 |  |
|  | rs10757274 | rs1333047 |  |
|  | rs10757278 | rs1333048 |  |
|  | rs1333042 | rs1537370 |  |
|  | rs1333043 | rs1537371 |  |
|  | rs1333047 | rs1537373 |  |
|  | rs1333048 | rs1556516 |  |
|  | rs1412834 | rs2891168 |  |
|  | rs1537370 | rs4977574 |  |
|  | rs1537371 | rs4977575 |  |
|  | rs1537372 | rs6475606 |  |
|  | rs1537373 | rs7859362 |  |
|  | rs1537374 | rs7859727 |  |
|  | rs1537375 |  |  |
|  | rs1556516 |  |  |
|  | rs2383206 |  |  |
|  | rs2383207 |  |  |
|  | rs2891168 |  |  |
|  | rs4977574 |  |  |
|  | rs4977575 |  |  |
|  | rs6475606 |  |  |
|  | rs7341786 |  |  |
|  | rs7859362 |  |  |
|  | rs7859727 |  |  |
|  | rs944797 |  |  |
|  | rs9632884 |  |  |
|  | rs9632885 |  |  |
|  |  |  |  |
| ***REST-NOA1*/rs17087335** | rs17087335 | rs17087335 | rs17087335 |
|  | rs17081935 | rs17081935 |  |
|  | rs7687767 | rs7687767 |  |
|  | rs2227901 | rs2227901 |  |
|  | rs3796529 | rs3796529 |  |
|  | rs781663 | rs781663 |  |
|  |  |  |  |
| ***NOS3/*rs3918226^a,b,c,d,e^** |  |  |  |
|  |  |  |  |
| ***SWAP70*/rs10840293^a,b^** | rs10840293 | rs10840293 | rs10840293 |
|  | rs93139 | rs93139 |  |
|  | rs93138 | rs93138 |  |
|  | rs360136 | rs360136 |  |
|  | rs360137 | rs360137 |  |
|  | rs173396 | rs173396 |  |
|  | rs360157 | rs360157 |  |
|  | rs378825 | rs378825 |  |
|  | rs360158 |  |  |
|  |  |  |  |
| ***SMAD3*/rs56062135^a,b^** | rs56062135 | rs56062135 | rs56062135 |
|  | rs16950687 | rs16950687 |  |
|  | rs7174445 | rs7174445 |  |
|  | rs7173698 | rs7173698 |  |
|  | rs2033784 | rs2033784 |  |
|  | rs8032739 | rs8032739 |  |
|  | rs1866316 | rs1866316 |  |
|  | rs17228058 | rs17228058 |  |
|  | rs2278546 | rs2278546 |  |
|  | rs17293632 | rs17293632 |  |
|  |  |  |  |
| ***MFGE8-ABHD2*/rs8042271^a,b,c,d,e^** |  |  |  |
|  |  |  |  |
| ***BCAS3/*rs7212798^a,b,c,d,e^** |  |  |  |
|  |  |  |  |
| ***PMAIP1-MC4R***  **/rs663129** | rs663129 | rs663129 | rs663129 |
|  | rs571312 | rs571312 |  |
|  | rs523288 | rs523288 |  |
|  | rs538656 | rs538656 |  |
|  | rs10871777 | rs10871777 |  |
|  | rs476828 | rs476828 |  |
|  | rs11152213 | rs11152213 |  |
|  | rs6567160 |  |  |
|  | rs2168711 |  |  |
|  | rs17782313 |  |  |
|  |  |  |  |
| ***POM121L9P-ADORA2A***  **/rs180803^a,b,c,d^**^,e^ |  |  |  |
|  |  |  |  |
| ***KSR2*/rs11830157** | rs11830157 | rs11830157 | rs11830157 |
|  |  |  |  |
| ***ZNF507-LOC400684/*rs12976411** | rs12976411 | rs12976411 | rs12976411 |
|  | rs16966931 | rs12985626 |  |
|  | rs12985626 |  |  |

**HapMap3 Warnings:**

a. No Query SNP in HapMap3_*r2*

b. No matching proxy SNP

c. No LD data is available in HapMap3_*r2*, panel CEU

**1000 genome Warnings:**

| d. No LD data is available in 1000 Genomes Pilot1, panel CEU |
| --- |
| e. Query SNP not in 1000Genomes Pilot1 |
