# Supplementary material for: Proficiency of data interpretation: identification of signaling SNPs/specific loci for coronary artery disease
Source: Database (Oxford). 2017 Oct 31;2017:bax078. doi: 10.1093/database/bax078 (PMC5737196; doi:10.1093/database/bax078)
Supplement: Supplementary Table 3 [file bax078_supp_table_s3.docx]

**Table S3. RegulomeDB scores and coordinates for all associated SNPs and SNPs in LD (*r^2^* ≥0.80)**

| **Coordinate(0-based)** | **dbSNP ID** | **RegulomeDB score** |
| --- | --- | --- |
| chr15:91429041 | rs1894401 | 1b |
| chr2:85808736 | rs1009 | 1b |
| **chr10:91005853** | **rs2246833** | **1b** |
| chr10:91002926 | rs1412444 | 1d |
| chr10:104616662 | rs4409766 | 1d |
| hr17:47038470 | rs4794004 | 1d |
| chr4:57824931 | rs7687767 | 1d |
| chr10:104873760 | rs9633712 | 1e |
| chr10:104721125 | rs10883808 | 1f |
| chr10:104871278 | rs10883832 | 1f |
| chr2:85794296 | rs10187424 | 1f |
| chr10:104660003 | rs11191454 | 1f |
| chr10:104723619 | rs11191479 | 1f |
| chr10:104764270 | rs11191499 | 1f |
| chr10:104773363 | rs11191514 | 1f |
| chr10:104776526 | rs11191515 | 1f |
| chr10:104846177 | rs11191548 | 1f |
| chr10:104864613 | rs11191557 | 1f |
| chr10:104864677 | rs11191558 | 1f |
| chr10:104906210 | rs11191580 | 1f |
| chr10:104913652 | rs11191582 | 1f |
| chr17:47028333 | rs11079844 | 1f |
| **chr7:129663495** | **rs11556924** | **1f** |
| chr10:104685298 | rs12411886 | 1f |
| chr10:104856161 | rs12412038 | 1f |
| chr10:104871203 | rs12413046 | 1f |
| **chr10:104719095** | **rs12413409** | **1f** |
| chr10:104681142 | rs17115213 | 1f |
| chr17:47005192 | rs15563 | 1f |
| chr2:85807081 | rs1348818 | 1f |
| chr17:47014126 | rs1994970 | 1f |
| chr17:47039131 | rs2291725 | 1f |
| **chr19:45395618** | **rs2075650** | **1f** |
| chr5:131723064 | rs17689550 | 1f |
| chr2:203880991 | rs2351524 | 1f |
| chr2:85742296 | rs2886722 | 1f |
| chr8:19813701 | rs271 | 1f |
| chr10:104825664 | rs3781285 | 1f |
| **chr17:46988596** | **rs46522** | **1f** |
| chr17:47008206 | rs4793992 | 1f |
| chr17:47047113 | rs3848460 | 1f |
| chr1:56996190 | rs4634932 | 1f |
| chr2:203880833 | rs4675310 | 1f |
| chr2:85805366 | rs3770098 | 1f |
| chr6:161152293 | rs4252126 | 1f |
| chr6:161154231 | rs4252135 | 1f |
| chr1:109818305 | rs629301 | 1f |
| chr1:109818529 | rs646776 | 1f |
| chr2:85774675 | rs6547621 | 1f |
| chr2:85783127 | rs6738645 | 1f |
| chr2:85803541 | rs6757263 | 1f |
| chr10:104835918 | rs943037 | 1f |
| **chr11:116648916** | **rs964184** | **1f** |
| chr11:9759712 | rs93138 | 1f |
| chr11:9773516 | rs360136 | 1f |
| chr4:57798188 | rs2227901 | 1f |
| chr2:85764959 | rs1446668 | 2a |
| chr10:104680136 | rs2297787 | 2a |
| chr17:46993232 | rs3744608 | 2a |
| chr1:56948289 | rs72664304 | 2a |
| chr15:67442595 | rs17293632 | 2a |
| chr10:104677125 | rs12221064 | 2b |
| chr10:104840966 | rs12219901 | 2b |
| chr17:47006492 | rs12601672 | 2b |
| chr1:109817589 | rs12740374 | 2b |
| chr10:44778545 | rs1746052 | 2b |
| chr10:91011457 | rs1332328 | 2b |
| chr10:91011680 | rs1332327 | 2b |
| chr1:222794090 | rs17163301 | 2b |
| chr10:91008878 | rs2250644 | 2b |
| **chr17:2117944** | **rs2281727** | **2b** |
| chr8:126478744 | rs2001844 | 2b |
| chr14:100116251 | rs28391527 | 2b |
| chr21:35593826 | rs28451064 | 2b |
| chr21:35644028 | rs28591415 | 2b |
| chr8:126476378 | rs2980856 | 2b |
| chr8:126478349 | rs2980853 | 2b |
| chr10:30316071 | rs3739998 | 2b |
| chr13:110960684 | rs4773143 | 2b |
| **chr13:110960711** | **rs4773144** | **2b** |
| chr14:100127439 | rs4624107 | 2b |
| chr8:19803092 | rs3779788 | 2b |
| chr10:44757106 | rs518594 | 2b |
| chr15:79152421 | rs5029904 | 2b |
| chr14:100125720 | rs7145262 | 2b |
| chr17:2098271 | rs7217687 | 2b |
| chr8:126479314 | rs6982636 | 2b |
| chr9:136154303 | rs649129 | 2b |
| chr2:203713279 | rs72934715 | 2b |
| chr2:203926270 | rs72934512 | 2b |
| chr2:203775474 | rs72936852 | 2b |
| chr1:154404335 | rs7549250 | 2b |
| chr1:154404379 | rs7549338 | 2b |
| chr1:154404405 | rs7553796 | 2b |
| chr17:2102452 | rs9908888 | 2b |
| chr15:67441996 | rs1866316 | 2b |
| chr15:67448898 | rs8032739 | 2b |
| chr9:22098573 | rs4977574 | 2c |
| chr10:104884207 | rs10883835 | 3a |
| chr10:44505533 | rs10793516 | 3a |
| chr17:2162118 | rs11078018 | 3a |
| chr17:2162120 | rs11078019 | 3a |
| chr17:47005587 | rs1057902 | 3a |
| chr6:35012301 | rs10456428 | 3a |
| chr10:44530977 | rs11238818 | 3a |
| hr1:154417043 | rs11265612 | 3a |
| **chr6:134214524** | **rs12190287** | **3a** |
| chr10:104928913 | rs12416331 | 3a |
| chr17:47029279 | rs12602933 | 3a |
| chr19:45387458 | rs12972156 | 3a |
| chr2:85773060 | rs12473819 | 3a |
| **chr10:44539912** | **rs2047009** | **3a** |
| chr10:91008872 | rs2250645 | 3a |
| chr17:47006949 | rs17635252 | 3a |
| chr2:85808572 | rs1972297 | 3a |
| **chr12:111884607** | **rs3184504** | **3a** |
| chr17:46977461 | rs2643361 | 3a |
| chr17:46977915 | rs2546491 | 3a |
| chr6:160883293 | rs3106162 | 3a |
| chr8:126480699 | rs2980882 | 3a |
| chr17:2130591 | rs4790887 | 3a |
| chr17:2162048 | rs422632 | 3a |
| chr6:161152239 | rs4252125 | 3a |
| chr10:44756702 | rs522293 | 3a |
| chr17:46985592 | rs61576918 | 3a |
| chr1:154405057 | rs4845619 | 3a |
| chr1:56991889 | rs56322312 | 3a |
| chr1:57016130 | rs56170783 | 3a |
| chr2:21290066 | rs580889 | 3a |
| chr2:21291311 | rs548145 | 3a |
| chr9:136149829 | rs532436 | 3a |
| chr1:154426096 | rs6689393 | 3a |
| chr1:56945665 | rs72664303 | 3a |
| chr2:203815739 | rs72926783 | 3a |
| **chr2:203855341** | **rs72932566** | **3a** |
| chr2:203673562 | rs72932755 | 3a |
| chr1:109817191 | rs7528419 | 3a |
| chr2:85772547 | rs7605975 | 3a |
| chr13:28982143 | rs7992068 | 3a |
| chr17:47018024 | rs8182364 | 3a |
| chr13:28984399 | rs9508026 | 3a |
| chr9:22112240 | rs7341786 | 3a |
| chr10:104594506 | rs1004467 | 4 |
| chr10:44516603 | rs10899971 | 4 |
| chr14:100111954 | rs10149871 | 4 |
| chr17:2143459 | rs10852932 | 4 |
| chr17:47005508 | rs1057897 | 4 |
| chr2:85769710 | rs1078004 | 4 |
| chr2:85788269 | rs10172544 | 4 |
| chr6:39174524 | rs10947787 | 4 |
| chr6:39174611 | rs10947788 | 4 |
| chr7:19055702 | rs10255384 | 4 |
| **chr19:11163600** | **rs1122608** | **4** |
| **chr9:136149710** | **rs115478735** | **4** |
| chr17:2098416 | rs11651451 | 4 |
| chr17:2119100 | rs11655813 | 4 |
| chr1:222906293 | rs12043288 | 4 |
| **chr6:35163019** | **rs12193946** | **4** |
| chr6:39166495 | rs12207689 | 4 |
| **chr6:39185242** | **rs12202900** | **4** |
| chr14:100131281 | rs12432679 | 4 |
| chr15:79151998 | rs12903542 | 4 |
| chr17:47031740 | rs12602746 | 4 |
| chr6:12911964 | rs12530250 | 4 |
| chr10:44777187 | rs1746050 | 4 |
| chr10:91002803 | rs1412445 | 4 |
| **chr15:91416549** | **rs17514846** | **4** |
| chr17:2095953 | rs1563966 | 4 |
| chr1:222828703 | rs17163363 | 4 |
| **chr1:56962820** | **rs17114036** | **4** |
| chr2:85809710 | rs1562323 | 4 |
| chr10:91005951 | rs2246828 | 4 |
| chr10:91010478 | rs2243547 | 4 |
| chr11:103668961 | rs2019090 | 4 |
| chr17:2126503 | rs216172 | 4 |
| chr17:2145328 | rs216218 | 4 |
| chr17:47022269 | rs2270574 | 4 |
| chr17:47039253 | rs2291726 | 4 |
| **chr2:145801460** | **rs2252641** | **4** |
| chr2:85742174 | rs2166529 | 4 |
| chr2:85758789 | rs2044474 | 4 |
| chr2:85767734 | rs2028900 | 4 |
| chr3:138119951 | rs2306374 | 4 |
| chr6:160872150 | rs1810126 | 4 |
| chr10:30323891 | rs2487928 | 4 |
| chr14:100130945 | rs34668196 | 4 |
| chr17:46975369 | rs318096 | 4 |
| chr2:85806067 | rs3731827 | 4 |
| chr10:104614349 | rs3824754 | 4 |
| chr10:44749170 | rs473501 | 4 |
| chr13:110960942 | rs3809346 | 4 |
| chr15:79141702 | rs4344704 | 4 |
| chr17:46993369 | rs4378658 | 4 |
| chr17:47009834 | rs4399576 | 4 |
| chr17:47047867 | rs3895874 | 4 |
| chr19:11161536 | rs3786722 | 4 |
| chr2:85764005 | rs3755014 | 4 |
| chr4:156631344 | rs3796592 | 4 |
| chr6:160853285 | rs388170 | 4 |
| chr6:161159005 | rs4252150 | 4 |
| chr6:161159015 | rs4252151 | 4 |
| chr6:35054671 | rs4276486 | 4 |
| chr6:39172528 | rs4711585 | 4 |
| chr10:44749707 | rs513391 | 4 |
| chr10:44755103 | rs579058 | 4 |
| chr10:44756235 | rs607760 | 4 |
| chr10:44756778 | rs605445 | 4 |
| chr10:44756893 | rs604674 | 4 |
| chr10:44757676 | rs492152 | 4 |
| chr17:47008756 | rs56046215 | 4 |
| chr19:11159888 | rs55948246 | 4 |
| chr1:154400014 | rs4845618 | 4 |
| chr1:154408339 | rs4845371 | 4 |
| chr1:222795568 | rs4846770 | 4 |
| chr6:161156666 | rs56093624 | 4 |
| chr6:161183454 | rs56262039 | 4 |
| chr6:39173117 | rs55818834 | 4 |
| chr9:136149398 | rs507666 | 4 |
| **chr9:136154167** | **rs579459** | **4** |
| chr10:44749210 | rs622472 | 4 |
| chr10:44755182 | rs622956 | 4 |
| chr10:44760032 | rs634963 | 4 |
| chr10:44761803 | rs694425 | 4 |
| **chr15:79141783** | **rs7173743** | **4** |
| chr15:91423542 | rs6224 | 4 |
| chr15:91428588 | rs7183988 | 4 |
| chr15:91428635 | rs7177338 | 4 |
| chr17:46986678 | rs62075824 | 4 |
| chr17:47026835 | rs62078370 | 4 |
| chr19:45394335 | rs71352238 | 4 |
| chr19:45414398 | rs72654473 | 4 |
| chr1:109817837 | rs660240 | 4 |
| chr1:154400319 | rs6687726 | 4 |
| chr1:56972352 | rs72664324 | 4 |
| chr1:56986977 | rs72664335 | 4 |
| chr1:56990752 | rs72664341 | 4 |
| chr2:203883192 | rs72932588 | 4 |
| chr2:203772983 | rs72936838 | 4 |
| chr2:204050473 | rs72936323 | 4 |
| chr2:203775711 | rs72936856 | 4 |
| chr10:104913939 | rs74233809 | 4 |
| chr17:2186099 | rs749240 | 4 |
| chr2:85738373 | rs7591175 | 4 |
| chr7:19059889 | rs7784712 | 4 |
| chr13:110960788 | rs7986871 | 4 |
| chr2:203830850 | rs79642273 | 4 |
| chr10:44516420 | rs9332446 | 4 |
| **chr13:111049622** | **rs9515203** | **4** |
| chr13:28984062 | rs9508025 | 4 |
| chr17:2121744 | rs9897624 | 4 |
| chr17:46969664 | rs9904645 | 4 |
| chr21:35595820 | rs9305545 | 4 |
| chr21:35606198 | rs9977419 | 4 |
| chr3:138117984 | rs9872754 | 4 |
| chr6:161171681 | rs9458022 | 4 |
| chr9:22088259 | rs10757272 | 4 |
| chr9:22098618 | rs2891168 | 4 |
| chr2:119481180 | rs11678748 | 5 |
| chr10:72851709 | rs7905985 | 5 |
| chr10:104721961 | rs78260931 | 5 |
| chr10:44538920 | rs7903121 | 5 |
| chr10:104841478 | rs10458729 | 5 |
| chr10:104883336 | rs1060240 | 5 |
| chr10:44501122 | rs10899963 | 5 |
| chr10:44509827 | rs10793517 | 5 |
| chr12:111910218 | rs10774625 | 5 |
| chr14:100115526 | rs10139462 | 5 |
| chr2:85762760 | rs10179195 | 5 |
| chr6:34707893 | rs10947525 | 5 |
| **chr6:39174921** | **rs10947789** | **5** |
| chr8:126495817 | rs10808546 | 5 |
| chr10:104766890 | rs11191505 | 5 |
| chr10:44505390 | rs11238808 | 5 |
| chr17:2138827 | rs11078883 | 5 |
| chr17:2170857 | rs1122645 | 5 |
| chr10:44535832 | rs11238822 | 5 |
| chr1:222834802 | rs1133220 | 5 |
| chr15:91428289 | rs11539637 | 5 |
| chr19:45396143 | rs11556505 | 5 |
| chr4:156630347 | rs115917428 | 5 |
| chr15:79143758 | rs11632020 | 5 |
| chr15:79169498 | rs11632720 | 5 |
| chr17:2175209 | rs11657644 | 5 |
| chr19:11159524 | rs12052058 | 5 |
| chr3:138087466 | rs1199338 | 5 |
| chr3:138088063 | rs1199337 | 5 |
| **chr6:35007635** | **rs12202483** | **5** |
| **chr6:35029418** | **rs12197124** | **5** |
| chr6:35047116 | rs12154140 | 5 |
| chr6:35049077 | rs12154189 | 5 |
| **chr6:35251316** | **rs12203818** | **5** |
| chr15:79136524 | rs12232282 | 5 |
| chr17:2124971 | rs1231209 | 5 |
| chr17:2125604 | rs1231206 | 5 |
| chr6:34898454 | rs12205331 | 5 |
| chr6:35008246 | rs12204265 | 5 |
| chr6:35009410 | rs12206298 | 5 |
| chr14:100112818 | rs12431602 | 5 |
| chr14:100135305 | rs12436072 | 5 |
| chr17:2112430 | rs12601834 | 5 |
| chr17:2156909 | rs12950555 | 5 |
| chr17:2160213 | rs12453323 | 5 |
| chr17:2160853 | rs12938295 | 5 |
| chr17:2197184 | rs12949991 | 5 |
| chr17:46994845 | rs12453374 | 5 |
| chr17:47010033 | rs12602179 | 5 |
| chr19:11167218 | rs12609863 | 5 |
| chr2:145824194 | rs13028626 | 5 |
| chr2:21265517 | rs12714264 | 5 |
| chr2:85787340 | rs12714145 | 5 |
| chr3:138117563 | rs13083299 | 5 |
| chr6:12927543 | rs12526453 | 5 |
| chr10:104591392 | rs17115100 | 5 |
| chr10:44512569 | rs1472168 | 5 |
| chr10:44775823 | rs1746048 | 5 |
| chr10:44776309 | rs1746049 | 5 |
| chr11:103696850 | rs1384705 | 5 |
| chr14:100133249 | rs17562391 | 5 |
| chr1:222806217 | rs17163345 | 5 |
| chr1:222837938 | rs17465982 | 5 |
| chr2:145843644 | rs13408842 | 5 |
| **chr2:85809988** | **rs1561198** | **5** |
| chr3:138070900 | rs13324341 | 5 |
| **chr6:35034799** | **rs17609940** | **5** |
| chr8:126486408 | rs17321515 | 5 |
| chr10:104630411 | rs17878846 | 5 |
| chr10:44500349 | rs1873756 | 5 |
| chr10:44539015 | rs1873757 | 5 |
| chr15:91428196 | rs2071382 | 5 |
| chr17:2096440 | rs2169357 | 5 |
| chr17:2134213 | rs216212 | 5 |
| chr17:2134224 | rs216213 | 5 |
| chr17:2147290 | rs216219 | 5 |
| chr17:2155700 | rs216222 | 5 |
| chr17:2155947 | rs216223 | 5 |
| chr17:2168508 | rs216176 | 5 |
| chr17:2168839 | rs216178 | 5 |
| chr17:2168877 | rs216179 | 5 |
| chr17:2187400 | rs216189 | 5 |
| chr17:2187456 | rs216190 | 5 |
| chr17:2195129 | rs216209 | 5 |
| chr17:2197636 | rs177567 | 5 |
| chr17:2205663 | rs2209073 | 5 |
| chr17:2205922 | rs2224770 | 5 |
| chr17:46977124 | rs2112617 | 5 |
| chr17:47011619 | rs17708633 | 5 |
| chr1:56939357 | rs1815487 | 5 |
| chr2:145815841 | rs1852683 | 5 |
| chr3:138124113 | rs2293251 | 5 |
| chr6:12907590 | rs2327620 | 5 |
| chr6:35169707 | rs2104332 | 5 |
| chr6:39182400 | rs2033226 | 5 |
| chr9:22010003 | rs2069416 | 5 |
| chr10:30306803 | rs2478839 | 5 |
| chr10:30311365 | rs2487927 | 5 |
| **chr10:30335121** | **rs2505083** | **5** |
| chr10:30335519 | rs2505084 | 5 |
| chr10:44549766 | rs2624695 | 5 |
| chr10:44551327 | rs2624694 | 5 |
| chr10:44566060 | rs2639463 | 5 |
| chr14:100116841 | rs2400762 | 5 |
| **chr14:100133941** | **rs2895811** | **5** |
| chr15:79134717 | rs28694044 | 5 |
| chr15:79134723 | rs28580532 | 5 |
| chr19:45395908 | rs34404554 | 5 |
| chr1:222793631 | rs2378584 | 5 |
| chr1:57008777 | rs2404715 | 5 |
| chr2:85809954 | rs2366639 | 5 |
| chr6:12907410 | rs2876301 | 5 |
| chr6:12907411 | rs2876302 | 5 |
| chr6:160849139 | rs2661839 | 5 |
| chr6:160850272 | rs3106164 | 5 |
| chr7:107245467 | rs35024078 | 5 |
| chr7:107249843 | rs35061190 | 5 |
| chr7:107249854 | rs35531458 | 5 |
| chr7:19046945 | rs2526619 | 5 |
| chr8:126477496 | rs2980854 | 5 |
| chr8:126477758 | rs2954019 | 5 |
| chr8:126481746 | rs2980875 | 5 |
| chr8:126482620 | rs2954022 | 5 |
| chr8:126485336 | rs2980860 | 5 |
| chr8:126488929 | rs2980871 | 5 |
| chr8:126491732 | rs2954031 | 5 |
| **chr9:22003222** | **rs3217992** | **5** |
| chr10:104595848 | rs3824755 | 5 |
| chr10:44749119 | rs474281 | 5 |
| chr17:2173496 | rs4790072 | 5 |
| chr17:2173498 | rs4790321 | 5 |
| chr17:2199748 | rs394752 | 5 |
| chr17:2199753 | rs375245 | 5 |
| chr17:2208898 | rs4523957 | 5 |
| chr17:46983390 | rs4294857 | 5 |
| chr17:46988528 | rs46521 | 5 |
| chr17:47019398 | rs4793995 | 5 |
| chr17:47035868 | rs4793605 | 5 |
| chr19:11168037 | rs3786728 | 5 |
| chr1:154410954 | rs4553185 | 5 |
| chr1:222834502 | rs4575092 | 5 |
| chr2:145818431 | rs4662414 | 5 |
| chr2:44074430 | rs4245791 | 5 |
| chr2:85764040 | rs3755015 | 5 |
| chr4:156638072 | rs3796587 | 5 |
| chr4:156642883 | rs3796581 | 5 |
| chr6:161137517 | rs4252107 | 5 |
| chr6:161137662 | rs4252109 | 5 |
| chr6:161175003 | rs4252181 | 5 |
| chr6:39186328 | rs4714223 | 5 |
| chr10:44490413 | rs4948591 | 5 |
| chr10:44511412 | rs4948815 | 5 |
| chr10:44511814 | rs4948818 | 5 |
| chr10:44740775 | rs498810 | 5 |
| chr10:44747058 | rs535176 | 5 |
| chr10:44752077 | rs559580 | 5 |
| chr10:44752117 | rs559469 | 5 |
| chr10:44752329 | rs535949 | 5 |
| chr10:44752557 | rs534079 | 5 |
| chr10:44753559 | rs503859 | 5 |
| chr10:44758196 | rs487465 | 5 |
| chr10:44764076 | rs493874 | 5 |
| chr14:100115607 | rs4905878 | 5 |
| chr14:100116585 | rs4990377 | 5 |
| chr17:2159501 | rs57307236 | 5 |
| chr17:46971139 | rs519537 | 5 |
| chr17:46998901 | rs58838744 | 5 |
| chr17:47019898 | rs4793998 | 5 |
| chr17:47022754 | rs4794000 | 5 |
| chr17:47029903 | rs4794003 | 5 |
| **chr1:154422066** | **rs4845625** | **5** |
| chr1:56988550 | rs56348932 | 5 |
| chr9:136154866 | rs495828 | 5 |
| chr10:44490337 | rs7090343 | 5 |
| chr10:44746139 | rs683297 | 5 |
| chr10:44752522 | rs684521 | 5 |
| chr10:44752975 | rs671765 | 5 |
| chr10:44757750 | rs665855 | 5 |
| chr10:44770472 | rs642222 | 5 |
| chr12:111932799 | rs7137828 | 5 |
| chr12:112007755 | rs653178 | 5 |
| chr14:100113287 | rs7146731 | 5 |
| chr14:100141688 | rs7158073 | 5 |
| chr15:79128888 | rs7168915 | 5 |
| chr17:2100511 | rs6503324 | 5 |
| chr17:2165339 | rs7213232 | 5 |
| chr17:46983170 | rs62075820 | 5 |
| chr17:47028988 | rs62078372 | 5 |
| chr17:47044462 | rs62078384 | 5 |
| chr17:47045008 | rs62078385 | 5 |
| chr1:154395945 | rs6689306 | 5 |
| chr1:154401971 | rs6694817 | 5 |
| chr1:154409099 | rs6667434 | 5 |
| chr1:56938217 | rs6588634 | 5 |
| chr2:203662196 | rs6705330 | 5 |
| chr2:203745326 | rs6722332 | 5 |
| chr2:203749438 | rs6738618 | 5 |
| **chr2:44073880** | **rs6544713** | **5** |
| chr2:85761278 | rs6705839 | 5 |
| chr2:85761416 | rs6705971 | 5 |
| chr2:85763519 | rs6743030 | 5 |
| chr4:156665073 | rs6849302 | 5 |
| chr6:12892485 | rs6925904 | 5 |
| chr6:161196561 | rs62436702 | 5 |
| chr6:39188690 | rs6918122 | 5 |
| chr9:136153874 | rs651007 | 5 |
| chr9:136154999 | rs635634 | 5 |
| chr1:56963626 | rs72664318 | 5 |
| chr1:57004416 | rs72664354 | 5 |
| chr21:35633709 | rs7280612 | 5 |
| chr21:35637461 | rs7278845 | 5 |
| chr2:203796813 | rs72926767 | 5 |
| chr2:203798584 | rs72926770 | 5 |
| chr2:203826395 | rs72926793 | 5 |
| chr2:203837572 | rs72928609 | 5 |
| chr2:203846816 | rs72932557 | 5 |
| chr2:203859390 | rs72932572 | 5 |
| chr2:203866856 | rs72932575 | 5 |
| chr2:203639394 | rs72932707 | 5 |
| chr2:203639500 | rs72932709 | 5 |
| chr2:203639845 | rs72932711 | 5 |
| chr2:203642243 | rs72932716 | 5 |
| chr2:203680953 | rs72932770 | 5 |
| chr2:203685727 | rs72932774 | 5 |
| chr2:203688299 | rs72932776 | 5 |
| chr2:203689913 | rs72932777 | 5 |
| chr2:203707751 | rs72934706 | 5 |
| chr2:203707877 | rs72934707 | 5 |
| chr2:203709775 | rs72934710 | 5 |
| chr2:203884307 | rs72932590 | 5 |
| chr2:203933338 | rs72934514 | 5 |
| chr2:203939639 | rs72934519 | 5 |
| chr2:203968972 | rs72934535 | 5 |
| chr2:203969503 | rs72934537 | 5 |
| chr2:203980032 | rs72934546 | 5 |
| chr2:203990788 | rs72934556 | 5 |
| chr2:204009056 | rs72934583 | 5 |
| chr2:204040295 | rs72936309 | 5 |
| chr2:204056677 | rs72936332 | 5 |
| chr19:11174741 | rs73013198 | 5 |
| chr19:11179708 | rs73013202 | 5 |
| chr2:203773685 | rs72936842 | 5 |
| chr2:203774733 | rs72936846 | 5 |
| chr2:203774747 | rs72936847 | 5 |
| chr2:203786811 | rs72936862 | 5 |
| chr2:203787119 | rs72936866 | 5 |
| chr2:203789678 | rs72936870 | 5 |
| chr10:44500806 | rs7478408 | 5 |
| chr2:203692759 | rs75166090 | 5 |
| chr2:203698299 | rs75324925 | 5 |
| chr2:203806723 | rs75869289 | 5 |
| chr6:12909873 | rs7454157 | 5 |
| chr10:104666756 | rs77180047 | 5 |
| chr10:104794085 | rs76752100 | 5 |
| chr10:104945822 | rs77420391 | 5 |
| **chr4:156635308** | **rs7692387** | **5** |
| chr10:104682601 | rs77602510 | 5 |
| chr6:12908407 | rs7760527 | 5 |
| chr7:19040330 | rs7792656 | 5 |
| chr10:104877301 | rs79082900 | 5 |
| chr10:104940945 | rs79237883 | 5 |
| chr13:28988536 | rs8002951 | 5 |
| chr2:203740937 | rs79539678 | 5 |
| chr15:91422542 | rs8039305 | 5 |
| chr17:2180822 | rs8065650 | 5 |
| chr17:2180967 | rs8066372 | 5 |
| chr21:35651289 | rs8132042 | 5 |
| chr6:35031440 | rs820082 | 5 |
| chr13:28976388 | rs9513097 | 5 |
| chr17:2115921 | rs9895551 | 5 |
| chr17:2121565 | rs9897160 | 5 |
| chr17:2126002 | rs9893573 | 5 |
| chr17:2133204 | rs9896535 | 5 |
| chr17:2156916 | rs9899193 | 5 |
| chr17:2161632 | rs9909895 | 5 |
| chr17:46972864 | rs832410 | 5 |
| chr17:47003220 | rs957557 | 5 |
| chr17:47046275 | rs937301 | 5 |
| **chr21:35599127** | **rs9982601** | **5** |
| chr21:35600504 | rs9980618 | 5 |
| chr21:35618637 | rs9978407 | 5 |
| chr3:138108351 | rs9848655 | 5 |
| chr3:138111750 | rs9864898 | 5 |
| **chr3:138122121** | **rs9818870** | **5** |
| chr6:160867158 | rs9355288 | 5 |
| chr6:161144799 | rs9458016 | 5 |
| chr6:161156295 | rs9458020 | 5 |
| chr11:9754220 | rs360157 | 5 |
| chr11:9759917 | rs173396 | 5 |
| chr11:9766931 | rs378825 | 5 |
| chr11:9773567 | rs360137 | 5 |
| chr15:67449659 | rs2033784 | 5 |
| chr15:67450304 | rs17228058 | 5 |
| chr15:67450892 | rs7173698 | 5 |
| chr15:67451214 | rs7174445 | 5 |
| chr15:67464012 | rs16950687 | 5 |
| chr15:67466601 | rs2278546 | 5 |
| chr18:57848530 | rs2168711 | 5 |
| chr18:57850421 | rs538656 | 5 |
| chr4:57781753 | rs781663 | 5 |
| chr4:57797413 | rs3796529 | 5 |
| chr9:22081396 | rs10116277 | 5 |
| chr9:22084309 | rs1537370 | 5 |
| chr9:22115285 | rs944797 | 5 |
| chr5:35873076 | rs11567758 | 6 |
| chr1:18310305 | rs11586673 | 6 |
| chr15:71773513 | rs11636588 | 6 |
| chrX:38919013 | rs7877578 | 6 |
| chr5:130865764 | rs1141093 | 6 |
| chr10:104689664 | rs10509759 | 6 |
| chr10:104713075 | rs10883806 | 6 |
| chr10:104739178 | rs10883815 | 6 |
| chr10:44496984 | rs10793515 | 6 |
| chr10:44523503 | rs10899973 | 6 |
| chr14:100117638 | rs10145905 | 6 |
| chr17:46968007 | rs1008834 | 6 |
| chr2:145814377 | rs10192407 | 6 |
| chr2:145834468 | rs1106909 | 6 |
| chr2:85794414 | rs10198569 | 6 |
| chr2:85808870 | rs1058588 | 6 |
| chr2:85808981 | rs1010 | 6 |
| chr7:19042748 | rs10245779 | 6 |
| chr9:22025492 | rs10738604 | 6 |
| chr10:104604915 | rs11191416 | 6 |
| chr10:104637507 | rs11191434 | 6 |
| chr10:104652322 | rs11191447 | 6 |
| chr10:104659851 | rs11191453 | 6 |
| chr10:104707015 | rs11191472 | 6 |
| chr10:104765493 | rs11191502 | 6 |
| chr10:104784917 | rs11191519 | 6 |
| chr10:104806897 | rs11191531 | 6 |
| chr10:104824386 | rs11191543 | 6 |
| chr10:104857522 | rs11191555 | 6 |
| chr10:104869037 | rs11191560 | 6 |
| chr10:104929715 | rs11191587 | 6 |
| chr10:104939214 | rs11191593 | 6 |
| chr17:2109108 | rs11078865 | 6 |
| chr17:2189323 | rs11078024 | 6 |
| chr10:104741030 | rs112390216 | 6 |
| chr2:203697091 | rs114520702 | 6 |
| chr2:203715513 | rs114372659 | 6 |
| chr2:203725676 | rs115130739 | 6 |
| chr2:203811911 | rs114604411 | 6 |
| chr2:203690046 | rs115628302 | 6 |
| chr2:204121144 | rs115600411 | 6 |
| chr2:203725677 | rs115827549 | 6 |
| chr15:79142306 | rs11639335 | 6 |
| chr2:203811901 | rs116773016 | 6 |
| hr4:156646339 | rs11724647 | 6 |
| chr4:156663103 | rs11721947 | 6 |
| chr15:79140837 | rs11857877 | 6 |
| chr17:47026989 | rs118135644 | 6 |
| chr7:19031934 | rs11984041 | 6 |
| chr10:104665266 | rs12221193 | 6 |
| chr10:104811202 | rs12219027 | 6 |
| chr10:104851888 | rs12217501 | 6 |
| chr10:104901490 | rs12220375 | 6 |
| chr10:104957628 | rs12414028 | 6 |
| chr17:17544703 | rs12449964 | 6 |
| chr17:2123199 | rs12602764 | 6 |
| chr17:2157773 | rs12943566 | 6 |
| chr17:2194873 | rs12941836 | 6 |
| chr1:109822142 | rs1277930 | 6 |
| chr2:145844224 | rs12618916 | 6 |
| chr3:138096096 | rs12695685 | 6 |
| chr4:156672737 | rs12502903 | 6 |
| chr10:104851300 | rs17094683 | 6 |
| chr10:44779077 | rs1657345 | 6 |
| chr13:28968467 | rs1555641 | 6 |
| chr17:2097482 | rs1532292 | 6 |
| chr17:2196904 | rs170045 | 6 |
| chr1:222820789 | rs17163360 | 6 |
| **chr1:222823528** | **rs17465637** | **6** |
| chr1:222899426 | rs17532708 | 6 |
| chr2:85759126 | rs17026396 | 6 |
| chr6:12908746 | rs13197912 | 6 |
| chr6:134226146 | rs162185 | 6 |
| chr6:161139856 | rs13231 | 6 |
| chr6:161145175 | rs1321197 | 6 |
| chr7:107225042 | rs13234124 | 6 |
| chr10:91004885 | rs2246942 | 6 |
| chr13:28963675 | rs2296284 | 6 |
| chr17:2172415 | rs216180 | 6 |
| chr17:2173922 | rs216183 | 6 |
| chr17:2188638 | rs216191 | 6 |
| chr17:2203452 | rs216193 | 6 |
| chr17:46967787 | rs1985785 | 6 |
| chr1:222832294 | rs2291834 | 6 |
| chr2:145811835 | rs1852685 | 6 |
| chr2:145821596 | rs1830319 | 6 |
| chr3:138121197 | rs2279241 | 6 |
| chr3:138122476 | rs2291127 | 6 |
| chr4:156638572 | rs2306556 | 6 |
| chr6:161146703 | rs1897108 | 6 |
| chr6:161154716 | rs1972748 | 6 |
| **chr7:19036774** | **rs2023938** | **6** |
| chr7:19037050 | rs2023937 | 6 |
| chr10:104942243 | rs34747231 | 6 |
| chr10:30317948 | rs2478835 | 6 |
| chr10:44558308 | rs2804029 | 6 |
| chr10:44560318 | rs2818904 | 6 |
| chr10:44750392 | rs2576354 | 6 |
| chr10:44760289 | rs2505732 | 6 |
| chr17:46989353 | rs318093 | 6 |
| chr17:46991751 | rs318090 | 6 |
| chr1:222816355 | rs35626308 | 6 |
| chr1:222850280 | rs3002126 | 6 |
| chr2:145832248 | rs2890769 | 6 |
| chr2:145832257 | rs2381687 | 6 |
| chr2:85790315 | rs35565292 | 6 |
| chr2:85792383 | rs35215812 | 6 |
| chr3:138095524 | rs2347252 | 6 |
| **chr5:131667352** | **rs273909** | **6** |
| chr6:12919866 | rs2876303 | 6 |
| chr6:160872651 | rs3088442 | 6 |
| chr8:126488234 | rs2980868 | 6 |
| chr8:126488249 | rs2980869 | 6 |
| chr10:104638479 | rs3740390 | 6 |
| chr12:111904370 | rs4766578 | 6 |
| chr15:79138477 | rs4420501 | 6 |
| chr15:79138564 | rs4438276 | 6 |
| chr15:79140691 | rs4567668 | 6 |
| chr17:2190766 | rs441750 | 6 |
| chr17:2190999 | rs404392 | 6 |
| chr17:2191259 | rs403553 | 6 |
| chr17:2191959 | rs432200 | 6 |
| chr17:2194027 | rs452363 | 6 |
| chr17:46996582 | rs4255820 | 6 |
| chr17:47001800 | rs4793991 | 6 |
| **chr19:45415639** | **rs445925** | **6** |
| chr1:222802802 | rs3748626 | 6 |
| chr2:203734364 | rs3845800 | 6 |
| chr2:44092065 | rs4603816 | 6 |
| chr6:12915416 | rs4711863 | 6 |
| chr6:161127124 | rs4252066 | 6 |
| chr6:161128921 | rs4252076 | 6 |
| chr6:161132416 | rs4252082 | 6 |
| chr6:161132724 | rs4252086 | 6 |
| chr6:161133243 | rs4252087 | 6 |
| chr6:161133684 | rs4252093 | 6 |
| chr6:161143375 | rs4252117 | 6 |
| **chr6:161143607** | **rs4252120** | **6** |
| chr6:161153079 | rs4252130 | 6 |
| chr6:161153526 | rs4252134 | 6 |
| chr6:161161558 | rs4252165 | 6 |
| chr10:44489654 | rs4948590 | 6 |
| **chr10:44753866** | **rs501120** | **6** |
| chr10:44758586 | rs573141 | 6 |
| chr10:44760472 | rs479596 | 6 |
| chr10:44762583 | rs528668 | 6 |
| chr10:44771665 | rs552794 | 6 |
| chr17:2114062 | rs59428454 | 6 |
| chr17:46982326 | rs62075818 | 6 |
| chr1:109822165 | rs599839 | 6 |
| chr1:56970417 | rs56186267 | 6 |
| chr2:21288225 | rs563290 | 6 |
| chr2:44096769 | rs4952688 | 6 |
| chr2:44097366 | rs4953026 | 6 |
| chr4:156633443 | rs56329057 | 6 |
| chr9:136151805 | rs600038 | 6 |
| chr10:44494657 | rs7091447 | 6 |
| chr10:44745846 | rs684666 | 6 |
| chr10:44745949 | rs684196 | 6 |
| chr10:44753374 | rs670056 | 6 |
| chr17:2136064 | rs7217226 | 6 |
| chr17:46996431 | rs62075838 | 6 |
| chr17:46996433 | rs62075839 | 6 |
| chr1:222823742 | rs67180937 | 6 |
| chr1:56940913 | rs6588635 | 6 |
| **chr2:203745884** | **rs6725887** | **6** |
| chr2:85755927 | rs6547620 | 6 |
| chr2:85756374 | rs6714709 | 6 |
| chr2:85802133 | rs6547624 | 6 |
| chr6:12902289 | rs6911226 | 6 |
| chr6:12902440 | rs6915983 | 6 |
| chr6:12934301 | rs6458545 | 6 |
| chr6:161134939 | rs62439805 | 6 |
| chr6:35003152 | rs62402708 | 6 |
| chr7:107264393 | rs71566736 | 6 |
| chr2:203801225 | rs72926771 | 6 |
| chr2:203808834 | rs72926781 | 6 |
| chr2:203822890 | rs72926791 | 6 |
| chr2:203827479 | rs72926796 | 6 |
| chr2:203836506 | rs72928608 | 6 |
| chr2:203839627 | rs72928613 | 6 |
| chr2:203840972 | rs72928620 | 6 |
| chr2:203842660 | rs72932553 | 6 |
| chr2:203847416 | rs72932560 | 6 |
| chr2:203848401 | rs72932561 | 6 |
| chr2:203861112 | rs72932573 | 6 |
| chr2:203866335 | rs72932574 | 6 |
| chr2:203647645 | rs72932723 | 6 |
| chr2:203649340 | rs72932725 | 6 |
| chr2:203650409 | rs72932731 | 6 |
| chr2:203661047 | rs72932741 | 6 |
| chr2:203662887 | rs72932745 | 6 |
| chr2:203670121 | rs72932753 | 6 |
| chr2:203679305 | rs72932767 | 6 |
| chr2:203682303 | rs72932772 | 6 |
| chr2:203697277 | rs72932781 | 6 |
| chr2:203698469 | rs72932784 | 6 |
| chr2:203698736 | rs72932786 | 6 |
| chr2:203701003 | rs72932793 | 6 |
| chr2:203740860 | rs72934738 | 6 |
| chr2:203746815 | rs72934749 | 6 |
| chr2:203747521 | rs72934751 | 6 |
| chr2:203750271 | rs72934753 | 6 |
| chr2:203755742 | rs72934760 | 6 |
| chr2:203758395 | rs72934763 | 6 |
| chr2:203758550 | rs72934765 | 6 |
| chr2:203769802 | rs72936830 | 6 |
| chr2:203773236 | rs72936839 | 6 |
| chr2:203927586 | rs72934513 | 6 |
| chr2:203995404 | rs72934563 | 6 |
| chr2:204005071 | rs72934573 | 6 |
| chr2:204017936 | rs72934589 | 6 |
| chr2:204031136 | rs72934601 | 6 |
| chr2:204036356 | rs72936304 | 6 |
| chr2:204054140 | rs72936326 | 6 |
| chr2:203790888 | rs72936872 | 6 |
| chr2:203794438 | rs72936882 | 6 |
| chr2:204121062 | rs72938315 | 6 |
| chr10:104897900 | rs732998 | 6 |
| chr2:203685118 | rs75141346 | 6 |
| chr2:203831251 | rs74421437 | 6 |
| chr10:104873252 | rs77055135 | 6 |
| chr10:30333622 | rs765906 | 6 |
| chr2:203751980 | rs77268589 | 6 |
| chr2:203805551 | rs77230711 | 6 |
| chr2:203814693 | rs76298043 | 6 |
| chr6:12891300 | rs7750679 | 6 |
| chr7:19059426 | rs7783974 | 6 |
| chr10:104748008 | rs77860422 | 6 |
| chr2:203763075 | rs77931721 | 6 |
| hr2:145852984 | rs787429 | 6 |
| chr10:104684543 | rs78821730 | 6 |
| chr10:104793903 | rs79668541 | 6 |
| chr17:47014991 | rs80032154 | 6 |
| chr21:35607495 | rs8131284 | 6 |
| chr10:44518914 | rs955584 | 6 |
| **chr11:103660566** | **rs974819** | **6** |
| chr13:28976715 | rs9508023 | 6 |
| chr13:28988794 | rs9513105 | 6 |
| chr17:2101133 | rs9900379 | 6 |
| chr17:2103809 | rs9908972 | 6 |
| chr17:2164310 | rs9891227 | 6 |
| chr17:2177731 | rs9905529 | 6 |
| chr17:46982748 | rs9912829 | 6 |
| chr17:47016881 | rs9747646 | 6 |
| chr1:56965663 | rs9970807 | 6 |
| chr21:35633529 | rs973754 | 6 |
| chr3:138121508 | rs9851766 | 6 |
| chr6:12898883 | rs9395214 | 6 |
| **chr6:12901440** | **rs9369640** | **6** |
| chr6:12913151 | rs9381494 | 6 |
| chr6:12922534 | rs9381500 | 6 |
| chr6:12922651 | rs9369650 | 6 |
| chr6:161130929 | rs9458012 | 6 |
| chr6:161147367 | rs9456578 | 6 |
| chr6:161150250 | rs9458017 | 6 |
| chr6:161185379 | rs9456580 | 6 |
| chr18:57829134 | rs6567160 | 6 |
| chr18:57839768 | rs571312 | 6 |
| chr18:57848368 | rs523288 | 6 |
| chr18:57851096 | rs17782313 | 6 |
| chr18:57851762 | rs10871777 | 6 |
| chr19:32828992 | rs12985626 | 6 |
| chr19:32829215 | rs16966931 | 6 |
| chr19:32882019 | rs12976411 | 6 |
| chr6:160961136 | rs3798220 | 6 |
| chr9:22072263 | rs10757269 | 6 |
| chr9:22088093 | rs10738607 | 6 |
| chr9:22112598 | rs10511701 | 6 |
| chr9:22116070 | rs1537375 | 6 |
| chr10:44489254 | rs10793511 | No Data |
| chr10:44501945 | rs10899965 | No Data |
| chr10:44515715 | rs10899970 | No Data |
| chr10:91007359 | rs1051338 | No Data |
| chr17:2097582 | rs1002135 | No Data |
| chr1:222825716 | rs10495198 | No Data |
| chr2:145820469 | rs10928240 | No Data |
| chr2:204048839 | rs10932008 | No Data |
| chr2:85761653 | rs10175792 | No Data |
| chr4:156645297 | rs10029150 | No Data |
| chr4:156676557 | rs10517620 | No Data |
| chr6:12923156 | rs1014342 | No Data |
| chr6:160839349 | rs10455782 | No Data |
| **chr7:107244544** | **rs10953541** | **No Data** |
| chr9:22014136 | rs10811641 | No Data |
| chr10:104625969 | rs11191425 | No Data |
| chr10:104850834 | rs11191551 | No Data |
| chr10:104898336 | rs11191575 | No Data |
| chr10:104959187 | rs11191607 | No Data |
| chr10:44527589 | rs11238817 | No Data |
| chr11:103693626 | rs11226029 | No Data |
| chr19:11164459 | rs112369586 | No Data |
| **chr1:55496038** | **rs11206510** | **No Data** |
| chr2:203825105 | rs114079739 | No Data |
| chr2:203718681 | rs114155121 | No Data |
| chr2:203721732 | rs114139737 | No Data |
| chr2:203823399 | rs114110842 | No Data |
| chr2:203831211 | rs114123510 | No Data |
| chr2:203769111 | rs114395475 | No Data |
| chr2:203787404 | rs114527590 | No Data |
| chr2:203795986 | rs114393235 | No Data |
| **chr2:203676104** | **rs115400054** | **No Data** |
| chr2:203721313 | rs115396314 | No Data |
| chr2:203771651 | rs114899426 | No Data |
| chr2:203821792 | rs114702158 | No Data |
| chr2:203829283 | rs115194657 | No Data |
| chr2:203893998 | rs115654617 | No Data |
| chr2:203943167 | rs115810193 | No Data |
| chr2:203744444 | rs115953525 | No Data |
| chr2:203751931 | rs116382857 | No Data |
| chr2:203819470 | rs116678869 | No Data |
| chr2:204196617 | rs116426890 | No Data |
| chr17:2176527 | rs11872068 | No Data |
| chr17:2176531 | rs11869805 | No Data |
| chr19:11159095 | rs12052201 | No Data |
| **chr6:39178782** | **rs12200013** | **No Data** |
| chr6:39180486 | rs12190363 | No Data |
| chr10:104769391 | rs12221335 | No Data |
| chr10:104851911 | rs12220743 | No Data |
| chr10:104931583 | rs12219304 | No Data |
| chr6:34644748 | rs12215331 | No Data |
| chr10:44529319 | rs12570314 | No Data |
| chr17:17537196 | rs12945496 | No Data |
| **chr17:17543721** | **rs12936587** | **No Data** |
| chr17:17547235 | rs12602348 | No Data |
| chr17:2112566 | rs12603057 | No Data |
| chr17:2119817 | rs12603592 | No Data |
| chr17:2159981 | rs12450354 | No Data |
| chr17:2194851 | rs12941621 | No Data |
| chr17:46967060 | rs12950328 | No Data |
| chr17:46994969 | rs12453394 | No Data |
| chr17:46997997 | rs12601072 | No Data |
| chr17:47017476 | rs12601858 | No Data |
| chr17:47034418 | rs12603969 | No Data |
| chr17:47034419 | rs12601955 | No Data |
| chr19:45387595 | rs12972970 | No Data |
| chr10:44513935 | rs1352999 | No Data |
| chr10:44773983 | rs1632484 | No Data |
| chr10:44774085 | rs1746047 | No Data |
| chr10:44781233 | rs1657344 | No Data |
| chr10:91003418 | rs1332329 | No Data |
| chr13:28962685 | rs17086617 | No Data |
| chr17:2132323 | rs143499 | No Data |
| chr17:2197501 | rs170044 | No Data |
| chr17:46991530 | rs170319 | No Data |
| chr1:222799624 | rs17163313 | No Data |
| chr1:222820638 | rs17163358 | No Data |
| chr1:222825087 | rs17011681 | No Data |
| chr1:222836995 | rs17465940 | No Data |
| chr1:56966349 | rs17114046 | No Data |
| chr2:203835176 | rs1541853 | No Data |
| chr3:138114290 | rs13096934 | No Data |
| chr6:12889003 | rs1332844 | No Data |
| chr10:30321597 | rs1887318 | No Data |
| chr10:44550714 | rs2128363 | No Data |
| chr10:91004915 | rs2246941 | No Data |
| chr11:103673276 | rs2128739 | No Data |
| chr17:2145089 | rs216217 | No Data |
| chr17:2148820 | rs216220 | No Data |
| chr17:2196087 | rs216206 | No Data |
| chr17:2196658 | rs216204 | No Data |
| chr17:2197386 | rs216202 | No Data |
| chr17:2198799 | rs216201 | No Data |
| chr17:2199845 | rs216200 | No Data |
| chr17:2201734 | rs216198 | No Data |
| chr17:47000109 | rs2088139 | No Data |
| chr1:222813724 | rs2133187 | No Data |
| chr1:222814441 | rs2133189 | No Data |
| chr1:222826480 | rs2291832 | No Data |
| chr2:145806095 | rs1976974 | No Data |
| chr2:145807414 | rs1852687 | No Data |
| chr2:145825554 | rs1830321 | No Data |
| chr6:12925935 | rs1953088 | No Data |
| chr6:160858187 | rs2292334 | No Data |
| **chr6:160863531** | **rs2048327** | **No Data** |
| chr6:160882028 | rs2063347 | No Data |
| chr6:160884274 | rs2063346 | No Data |
| chr6:161132803 | rs1853018 | No Data |
| chr6:35006408 | rs2091074 | No Data |
| chr6:35089810 | rs2077750 | No Data |
| chr10:104656038 | rs35125602 | No Data |
| chr10:44569664 | rs2804027 | No Data |
| chr10:44750361 | rs2576355 | No Data |
| chr11:103673293 | rs2839812 | No Data |
| chr17:2125443 | rs28524880 | No Data |
| chr17:46974733 | rs318095 | No Data |
| chr17:46976755 | rs28517720 | No Data |
| chr17:46991555 | rs318091 | No Data |
| chr17:46998117 | rs28409394 | No Data |
| chr19:45388129 | rs34342646 | No Data |
| chr1:222811406 | rs35700460 | No Data |
| chr21:35632169 | rs28593428 | No Data |
| chr2:145826053 | rs2381686 | No Data |
| chr2:145848069 | rs28709970 | No Data |
| chr2:203765755 | rs35212307 | No Data |
| chr2:21271706 | rs34722314 | No Data |
| chr2:21272895 | rs35913552 | No Data |
| chr3:138121919 | rs3732837 | No Data |
| chr6:12922688 | rs2327621 | No Data |
| chr6:12922733 | rs34343839 | No Data |
| chr6:160842536 | rs3123636 | No Data |
| chr6:161168948 | rs28402939 | No Data |
| chr7:107226155 | rs34523267 | No Data |
| chr7:107249019 | rs34273277 | No Data |
| chr8:126477475 | rs2980855 | No Data |
| chr8:126485293 | rs2954027 | No Data |
| **chr8:126490971** | **rs2954029** | **No Data** |
| chr8:19811896 | rs254 | No Data |
| chr8:19811900 | rs255 | No Data |
| chr8:19811966 | rs256 | No Data |
| **chr8:19813179** | **rs264** | **No Data** |
| chr9:136141869 | rs2519093 | No Data |
| chr17:2173183 | rs4790071 | No Data |
| chr17:2192084 | rs4790325 | No Data |
| chr17:47019596 | rs4793996 | No Data |
| chr17:47019671 | rs4793997 | No Data |
| chr19:11166475 | rs3786723 | No Data |
| chr2:203734865 | rs4510208 | No Data |
| chr2:44072575 | rs4299376 | No Data |
| chr6:12903434 | rs4714955 | No Data |
| chr6:12927844 | rs4714990 | No Data |
| chr6:160868653 | rs3918285 | No Data |
| chr6:160868667 | rs3918286 | No Data |
| chr6:161128263 | rs4252072 | No Data |
| chr6:161133543 | rs4252090 | No Data |
| chr6:161134272 | rs4252096 | No Data |
| chr6:161143189 | rs4252114 | No Data |
| chr10:104697515 | rs5011520 | No Data |
| chr10:44746394 | rs541483 | No Data |
| chr10:44749989 | rs510785 | No Data |
| chr10:44750208 | rs607609 | No Data |
| chr10:44750214 | rs607592 | No Data |
| chr10:44750668 | rs605425 | No Data |
| chr10:44753455 | rs504799 | No Data |
| chr10:44755445 | rs554568 | No Data |
| chr10:44755447 | rs554565 | No Data |
| chr10:44761072 | rs620356 | No Data |
| chr10:44761622 | rs607363 | No Data |
| chr10:44762685 | rs527785 | No Data |
| chr10:44773497 | rs494045 | No Data |
| chr17:2112719 | rs62069332 | No Data |
| chr17:46957986 | rs595767 | No Data |
| chr17:46999372 | rs60708039 | No Data |
| chr17:46999936 | rs55771415 | No Data |
| chr17:47017878 | rs55724082 | No Data |
| chr17:47026075 | rs58591767 | No Data |
| chr17:47026184 | rs59270107 | No Data |
| chr1:109821306 | rs583104 | No Data |
| **chr1:109821510** | **rs602633** | **No Data** |
| chr1:154406539 | rs59632925 | No Data |
| chr1:222797613 | rs4846384 | No Data |
| chr1:56952749 | rs55694910 | No Data |
| chr1:56986632 | rs55869368 | No Data |
| chr21:35602267 | rs60687229 | No Data |
| chr2:21281855 | rs481069 | No Data |
| **chr2:21286056** | **rs515135** | **No Data** |
| chr2:21288320 | rs562338 | No Data |
| chr2:21289431 | rs581411 | No Data |
| chr2:21294974 | rs541041 | No Data |
| chr2:85759493 | rs59877521 | No Data |
| chr4:156661062 | rs56256623 | No Data |
| chr10:104628233 | rs7098825 | No Data |
| chr10:44751909 | rs687175 | No Data |
| chr10:44760957 | rs620828 | No Data |
| chr17:2096579 | rs6503321 | No Data |
| chr17:2135979 | rs7212249 | No Data |
| chr17:2176963 | rs6502155 | No Data |
| chr17:47002388 | rs62075844 | No Data |
| chr17:47017175 | rs62075852 | No Data |
| chr1:56915592 | rs6421496 | No Data |
| chr2:203738663 | rs6723704 | No Data |
| chr2:203753015 | rs6435169 | No Data |
| chr2:21291528 | rs668948 | No Data |
| chr2:44096335 | rs6755809 | No Data |
| chr2:44096401 | rs6544717 | No Data |
| chr2:85755356 | rs6739015 | No Data |
| chr2:85757370 | rs6719046 | No Data |
| chr2:85757378 | rs6733913 | No Data |
| chr3:138052753 | rs6807945 | No Data |
| chr4:156664809 | rs6536087 | No Data |
| chr6:12902247 | rs62389955 | No Data |
| chr6:12923766 | rs62386818 | No Data |
| chr7:107259720 | rs68170813 | No Data |
| chr9:22048413 | rs7028268 | No Data |
| chr21:35621364 | rs7278204 | No Data |
| chr2:203798317 | rs72926769 | No Data |
| chr2:203804288 | rs72926772 | No Data |
| chr2:203805928 | rs72926779 | No Data |
| chr2:203811627 | rs72926782 | No Data |
| chr2:203816493 | rs72926786 | No Data |
| chr2:203818298 | rs72926787 | No Data |
| chr4:156639887 | rs72689147 | No Data |
| chr2:203827431 | rs72926794 | No Data |
| chr2:203827674 | rs72926798 | No Data |
| chr2:203828191 | rs72926799 | No Data |
| chr2:203829224 | rs72926800 | No Data |
| chr2:203830227 | rs72926802 | No Data |
| chr2:203832119 | rs72928605 | No Data |
| chr2:203838960 | rs72928610 | No Data |
| chr2:203845827 | rs72932554 | No Data |
| chr2:203845995 | rs72932556 | No Data |
| chr2:203847324 | rs72932558 | No Data |
| chr2:203847381 | rs72932559 | No Data |
| chr2:203869846 | rs72932583 | No Data |
| chr2:203646933 | rs72932720 | No Data |
| chr2:203647597 | rs72932722 | No Data |
| chr2:203649500 | rs72932727 | No Data |
| chr2:203654539 | rs72932737 | No Data |
| chr2:203663497 | rs72932746 | No Data |
| chr2:203667525 | rs72932752 | No Data |
| chr2:203674287 | rs72932759 | No Data |
| chr2:203678657 | rs72932763 | No Data |
| chr2:203679182 | rs72932765 | No Data |
| chr2:203695825 | rs72932780 | No Data |
| chr2:203699378 | rs72932789 | No Data |
| chr2:203700869 | rs72932791 | No Data |
| chr2:203706432 | rs72934704 | No Data |
| chr2:203709996 | rs72934711 | No Data |
| chr2:203711580 | rs72934714 | No Data |
| chr2:203737769 | rs72934729 | No Data |
| chr2:203739855 | rs72934732 | No Data |
| chr2:203739969 | rs72934734 | No Data |
| chr2:203740009 | rs72934735 | No Data |
| chr2:203740797 | rs72934737 | No Data |
| chr2:203741361 | rs72934740 | No Data |
| chr2:203744453 | rs72934745 | No Data |
| chr2:203756676 | rs72934762 | No Data |
| chr2:203758520 | rs72934764 | No Data |
| chr2:203766562 | rs72934767 | No Data |
| chr2:203771259 | rs72936834 | No Data |
| chr2:203916486 | rs72934505 | No Data |
| chr2:203925359 | rs72934510 | No Data |
| chr2:203937907 | rs72934518 | No Data |
| chr2:203975957 | rs72934545 | No Data |
| chr2:203983939 | rs72934550 | No Data |
| chr2:203984116 | rs72934551 | No Data |
| chr2:203987805 | rs72934554 | No Data |
| chr2:204019761 | rs72934591 | No Data |
| chr2:204064838 | rs72936348 | No Data |
| chr2:204070955 | rs72936353 | No Data |
| chr19:11183836 | rs73015007 | No Data |
| chr2:203783483 | rs72936860 | No Data |
| chr2:203788949 | rs72936869 | No Data |
| chr2:203791332 | rs72936873 | No Data |
| chr2:203791911 | rs72936875 | No Data |
| chr2:203792627 | rs72936879 | No Data |
| chr2:203794261 | rs72936881 | No Data |
| chr10:104655349 | rs74233296 | No Data |
| chr10:104769274 | rs74444347 | No Data |
| chr17:2157924 | rs7406247 | No Data |
| chr2:203673072 | rs74675536 | No Data |
| chr2:203753071 | rs7582720 | No Data |
| chr2:203757915 | rs7560547 | No Data |
| chr10:104636275 | rs77335224 | No Data |
| chr10:104793647 | rs75970938 | No Data |
| chr2:145811604 | rs7592365 | No Data |
| chr2:145836428 | rs7593336 | No Data |
| chr2:203664928 | rs76122535 | No Data |
| chr2:203724494 | rs7605484 | No Data |
| chr2:203850010 | rs76890136 | No Data |
| chr2:203865514 | rs76461893 | No Data |
| chr6:12934686 | rs7739181 | No Data |
| chr10:104776204 | rs77787671 | No Data |
| chr6:12900976 | rs7751826 | No Data |
| chr6:12908076 | rs7760016 | No Data |
| chr6:160840251 | rs7758229 | No Data |
| chr6:160865644 | rs7769879 | No Data |
| chr6:35008677 | rs7742443 | No Data |
| chr7:107243994 | rs7785962 | No Data |
| chr2:203663974 | rs78128841 | No Data |
| chr2:145852958 | rs787428 | No Data |
| chr2:203932175 | rs78907692 | No Data |
| chr10:104901030 | rs79993475 | No Data |
| chr13:28964324 | rs7983774 | No Data |
| chr2:203683989 | rs79633844 | No Data |
| chr17:2099930 | rs8076939 | No Data |
| chr17:2184554 | rs8077545 | No Data |
| chr17:2187930 | rs8074850 | No Data |
| chr21:35625112 | rs8131303 | No Data |
| chr2:203673071 | rs80087860 | No Data |
| chr6:12919988 | rs8180558 | No Data |
| chr10:44755405 | rs915083 | No Data |
| chr13:28967659 | rs9513095 | No Data |
| **chr13:28973620** | **rs9319428** | **No Data** |
| chr13:28973702 | rs9319429 | No Data |
| chr13:28991987 | rs9508029 | No Data |
| chr13:28993668 | rs9513106 | No Data |
| chr13:28993834 | rs9513107 | No Data |
| chr17:17531790 | rs9630706 | No Data |
| chr17:17546825 | rs9913096 | No Data |
| chr17:17548632 | rs9900673 | No Data |
| chr17:2129209 | rs9906500 | No Data |
| chr17:46978282 | rs962272 | No Data |
| chr17:46980317 | rs903567 | No Data |
| chr17:46997958 | rs9894239 | No Data |
| chr21:35596841 | rs9976596 | No Data |
| chr21:35641432 | rs9983490 | No Data |
| chr6:12894903 | rs9296512 | No Data |
| chr6:160856842 | rs9365164 | No Data |
| chr6:161153560 | rs9458019 | No Data |
| chr5:173243818 | rs11458630 | No Data |
| chr11:9753600 | rs360158 | No Data |
| chr11:9759607 | rs93139 | No Data |
| chr12:118265440 | rs11830157 | No Data |
| chr18:57838400 | rs663129 | No Data |
| chr18:57852586 | rs476828 | No Data |
| chr18:57852947 | rs11152213 | No Data |
| chr4:57823475 | rs17081935 | No Data |
| chr4:57838582 | rs17087335 | No Data |
| chr9:22081849 | rs6475606 | No Data |
| chr9:22115958 | rs2383207 | No Data |
| chr9:22125346 | rs1333048 | No Data |
| chr9:22125502 | rs1333049 | No Data |

GWAS significant SNPs are written in bold
