# Supplementary figures and images for: Proficiency of data interpretation: identification of signaling SNPs/specific loci for coronary artery disease
Source: Database (Oxford). 2017 Oct 31;2017:bax078. doi: 10.1093/database/bax078 (PMC5737196; doi:10.1093/database/bax078)

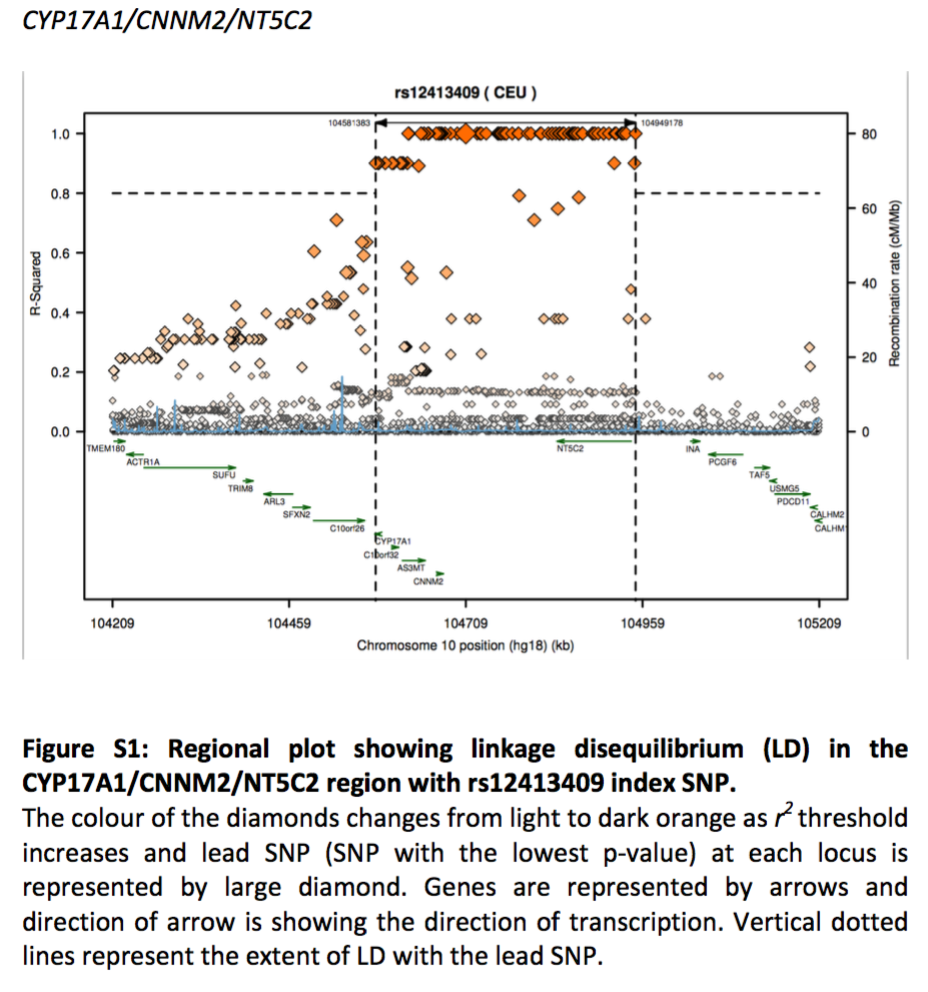

Supplement: Supplementary Figure 1 [file bax078_supp_figure_s1.png]
